# The archaeal RNA chaperone TRAM0076 shapes the transcriptome and optimizes the growth of *Methanococcus maripaludis*

**DOI:** 10.1371/journal.pgen.1008328

**Published:** 2019-08-12

**Authors:** Jie Li, Bo Zhang, Liguang Zhou, Lei Qi, Lei Yue, Wenting Zhang, Huicai Cheng, William B. Whitman, Xiuzhu Dong

**Affiliations:** 1 State Key Laboratory of Microbial Resources, Institute of Microbiology, Chinese Academy of Sciences, Beijing, PR China; 2 University of Chinese Academy of Sciences, Shijingshan District, Beijing, China; 3 Biology Institute, Hebei Academy of Sciences, Shijiazhuang, China; 4 Department of Microbiology, University of Georgia, Athens, Georgia, United States of America; Max-Planck-Institut fur terrestrische Mikrobiologie, GERMANY

## Abstract

TRAM is a conserved domain among RNA modification proteins that are widely distributed in various organisms. In Archaea, TRAM occurs frequently as a standalone protein with *in vitro* RNA chaperone activity; however, its biological significance and functional mechanism remain unknown. This work demonstrated that TRAM0076 is an abundant standalone TRAM protein in the genetically tractable methanoarcheaon *Methanococcus maripaludis*. Deletion of *MMP0076*, the gene encoding TRAM0076, markedly reduced the growth and altered transcription of 55% of the genome. Substitution mutations of Phe39, Phe42, Phe63, Phe65 and Arg35 in the recombinant TRAM0076 decreased the *in vitro* duplex RNA unfolding activity. These mutations also prevented complementation of the growth defect of the *MMP0076* deletion mutant, indicating that the duplex RNA unfolding activity was essential for its physiological function. A genome-wide mapping of transcription start sites identified many 5′ untranslated regions (5′UTRs) of 20–60 nt which could be potential targets of a RNA chaperone. TRAM0076 unfolded three representative 5′UTR structures *in vitro* and facilitated the *in vivo* expression of a mCherry reporter system fused to the 5′UTRs, thus behaving like a transcription anti-terminator. Flag-tagged-TRAM0076 co-immunoprecipitated a large number of cellular RNAs, suggesting that TRAM0076 plays multiple roles in addition to unfolding incorrect RNA structures. This work demonstrates that the conserved archaeal RNA chaperone TRAM globally affects gene expression and may represent a transcriptional element in ancient life of the RNA world.

## Author summary

RNAs frequently misfold into stable but biologically inactive structures especially under stresses, while RNA chaperones interact with various RNAs to prevent the structures that may cause premature transcriptional termination or pausing, and affect mRNA decay and translation. This work for the first time reports that an archaeal RNA chaperone TRAM0076 globally affects the transcription of methanogenic Archaea through posttranscriptional actions. TRAM0076 also binds many cellular mRNAs, possibly at the 5′-untranslated regions. This work uncovers an important regulatory element of ancient life in the RNA world.

## Introduction

RNA chaperones impact mRNA metabolism including transcript synthesis, processing and degradation as well as translation. They play an important role in controlling gene expression, especially in response to environmental perturbations such as cold shock [1]. The distinctive features of RNA chaperones include the absence of an energy requirement for activity, transient interactions with their RNA targets without obvious sequence specificity, and the ability to melt kinetically trapped RNA hairpin structures [2, 3]. These characteristics enable RNA chaperones to interact with many classes of RNAs and through lowering the energetic barriers to assist RNA folding into thermodynamically favorable conformations [3]. Extensively studied bacterial RNA chaperones include cold shock proteins (Csps), translation initiation factors (IFs), ribosomal proteins and the Sm-like protein Hfq. Csps are small proteins that affect cellular processes by remodeling RNAs for recycling or translation and preventing premature transcriptional termination via melting RNA hairpin structures, particularly at low temperatures [4–7]. Hfq is a global post-transcriptional regulator that impacts gene expression in a wide range of bacteria by facilitating annealing of small noncoding RNAs (sRNAs) and target mRNAs at two distinct binding faces so as to repress translation and/or accelerate mRNA decay [8, 9]. In contrast, only a few archaeal RNA chaperones have been reported, and little is known about their physiological functions.

In methanogens as well as other Archaea, transcription of messenger as well as stable RNAs employs a homolog of the eukaryotic RNA polymerase II [10]. Transcription initiation in Archaea requires two basal transcriptional factors, TATA-box binding protein (TBP) and transcription factor B (TFB) [11, 12], and a third conserved factor, transcription factor E (TFE) [13, 14]. However, transcriptional regulation in archaea is not well understood. Sigma factors, which are common in bacteria, are absent [15]. Some Archaea, such as haloarchaea, encode multiple basic transcription factors, different combinations of which could be used to achieve a certain level of differential transcription [16–18]. However, many methanogenic Archaea only possess single copies of the *tbp* and *tfb* genes, so this general mechanism of transcriptional regulation appears to be restricted to just a few groups. While homologs of some of the bacterial transcription regulatory proteins have been identified in methanogens, these genes are not abundant in many methanogens and archaea in general [19]. For instance, the genome of *Methanococcus maripaludis* S2 possesses only 29 homologs of bacterial transcription factors, and the function of most of these genes is unknown. This suggests that transcription regulation may not be prevalent in methanoarchaea. Noticeably, genome-wide transcription start site mapping indicates that long 5′ un-translation regions (5′UTR) are common in methanogenic archaea [20–22]. In bacteria, the 5′UTRs of mRNAs are often targeted by non-coding small RNAs, ribonucleases and RNA chaperones and are potential elements for RNA-based regulation of gene expression [23, 24]. The long 5′UTRs found in methanogens could act similarly [20, 21], and they might also misfold into stable but biologically inactive structures that need to be unfolded during active growth. However, RNA chaperones that interact with 5′UTRs to regulate gene expression in methanogens and Archaea have not been demonstrated.

TRAM is an archetype domain found in many proteins involved in RNA modifications, such as TRM2 methylases and methylthiotransferase RimO, as well as the translation initiation factor IF-2β subunit and ribosomal protein S2 [25]. Proteins containing TRAM domain are found in all the living organisms [26]. Distinctively, most archaea possess a small protein that contains a standalone TRAM domain with an OB (Oligonucleotide-Binding) fold [26], which is widely distributed in most archaeal phyla (S1 Fig). The *in vitro* studies indicated that these TRAM proteins exhibit typical properties of RNA chaperones. A cold-responsive standalone TRAM protein from the cold-tolerant methanogenic archaeon *Methanococcoides burtonii* called Ctr3 was reported to preferentially bind tRNAs and 5S rRNA [27]. The psychrophilic methanogen *Methanolobus psychrophilus* R15 possesses four TRAM proteins that can replace the cold shock proteins in *E*. *coli* and exhibit nonspecific RNA binding and duplex RNA melting activity *in vitro* [28]. However, the biological significance of these standalone archaeal TRAM proteins remains to be clarified.

In this study, *M*. *maripaludis* was selected to interrogate the biological significance of archaeal standalone TRAM proteins and their regulatory role in gene expression. *M*. *maripaludis* has only a single TRAM-encoding gene (*MMP0076*) and is genetic tractable. Through genetic, physiological, biochemical and transcriptomic studies, we demonstrate that the standalone TRAM protein MMP0076 (named TRAM0076 hereafter) from *M*. *maripaludis* acts as an RNA chaperone and is required for the normal expression of more than half of the genome and normal growth. TRAM0076 controls the expression of some mRNAs through unfolding the kinetically trapped RNA structures in the 5′UTR elements. Thus, the archaeal standalone TRAM protein appears to play a fundamental role in transcription.

## Results

### Deletion of the TRAM gene reduces growth of *M*. *maripaludis*

**Fig 1:**
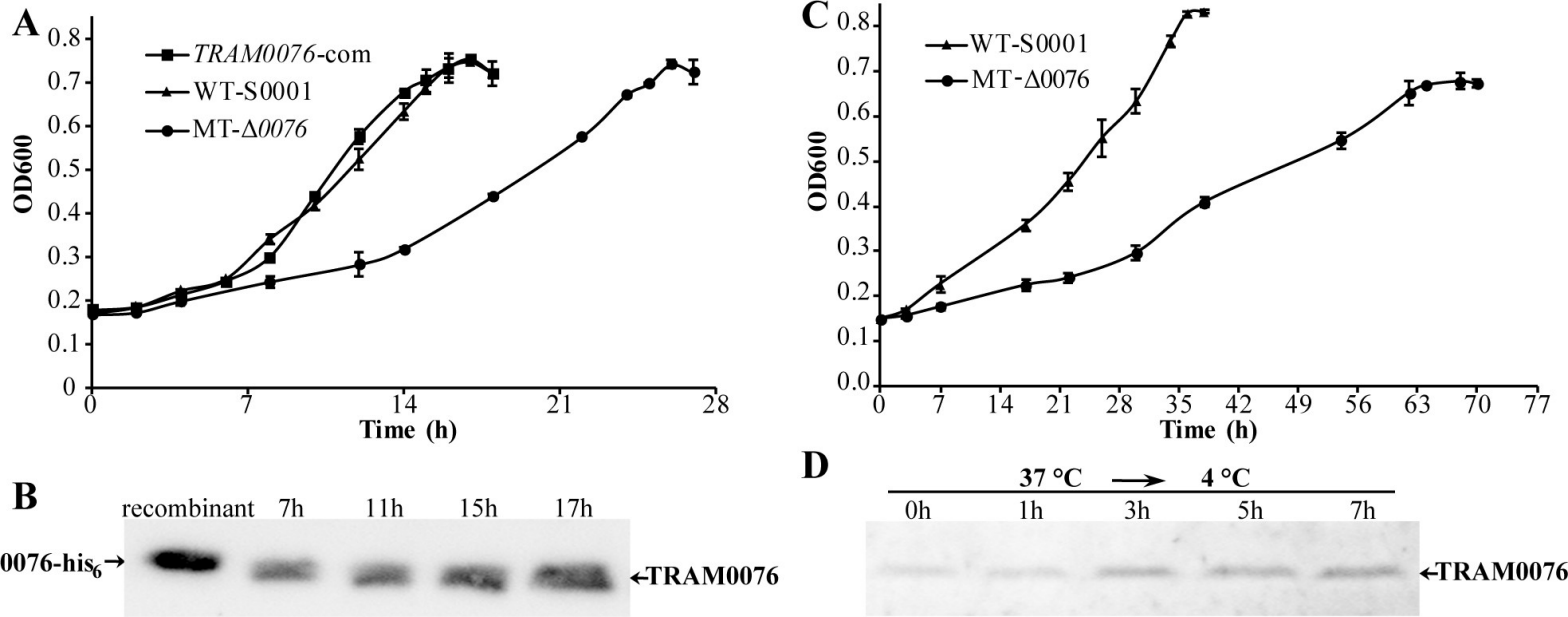
Deletion of the gene encoding TRAM0076 reduces the growth rate of *Methanococcus maripaludis*. Homologous recombination replaced the *MMP0076* gene of *M*. *maripaludis* S0001 with the *pac* cassette encoding puromycin resistance, and produced strain Δ*0076*. (A) Deletion of *MMP0076* significantly reduces the growth rate at 37°C from 0.15 ± 0.001 h^-1^ of the parental strain (WT-S0001) to 0.065 ± 0.003 h^-1^ of the mutant (MT-Δ*0076*). The *MMP0076* complementation (*MMP0076*-com) restored the growth of the mutant. Growth at 37°C was followed by monitoring OD600nm, and the average and standard deviation from triplicate cultures are shown. (B) Western blotting assays of the TRAM0076 abundance throughout the entire growth phase. Total cellular protein (7 μg) from the cultures at early (7h)-, middle (11h)- and late-exponential (15h), and stationary (17h) growth phases was used for Western blotting with the antibody against TRAM0076, and 9 ng of purified recombinant His_6_-tagged TRAM0076 protein was included as reference (recombinant). (C) Growth of the *MMP0076* deletion mutant (Δ*0076*) was also impaired at 22°C. The growth rates of *M*. *maripaludis* S0001 and the Δ*0076* mutant were 0.042 ± 0.002 h^-1^ and 0.023 ± 0.001 h^-1^, respectively. (D) Cold-shock induced expression of TRAM0076. A mid-exponential phase culture of strain S0001 grown at 37°C was incubated at 4°C for up to 7 hours. TRAM0076 protein abundance was assayed by Western blotting as in Fig 1B.

To examine the biological significance of TRAM proteins in Archaea, *MMP0076*, which encodes a standalone TRAM protein (TRAM0076), was replaced with the *pac* cassette for puromycin resistance in *M*. *maripaludis* S0001 as described by Tumbula *et al*. [29], resulting in the construction of the Δ*MMP0076*::*pac* deletion mutant (Δ*0076*). The growth rate of the mutant (0.065 ± 0.003 h^-1^ vs 0.15 ± 0.001 h^-1^ of strain S0001) was markedly reduced at the optimal growth temperature of 37°C. To confirm that the growth reduction phenotype was not due to a mutation at a second site, the Δ*0076* mutant was complemented by expressing TRAM0076 from the replicative plasmid pMEV2-*MMP0076* carrying the *MMP0076* gene (*MMP0076*-com strain). Growth of strain *MMP0076*-com was completely restored to wild-type levels (Fig 1A). These results indicate that TRAM0076 is necessary for optimal growth of *M*. *maripaludis*. Moreover, Western blotting indicated that the levels of TRAM0076 increased by about 50% in older cultures (Fig 1B). By reference to the recombinant purified protein (Fig 1B, lane recombinant), the cellular TRAM0076 level was estimated to account for about 0.05–0.1% of the total protein.

Growth of the Δ*0076* mutant at low temperatures was also examined. Compared to the parental strain S0001, the Δ*0076* mutant showed a similar reduction of the growth rate at 22°C as at 37°C (Fig 1C). Moreover, cold shock at 4°C led to an increase of the cellular TRAM0076 content by 50–80% (Fig 1D). These results suggest that while TRAM0076 is differentially expressed depending on the growth conditions, its function is not limited to withstanding cold stress.

### Deletion of the TRAM gene markedly changes the transcriptome

**Fig 2:**
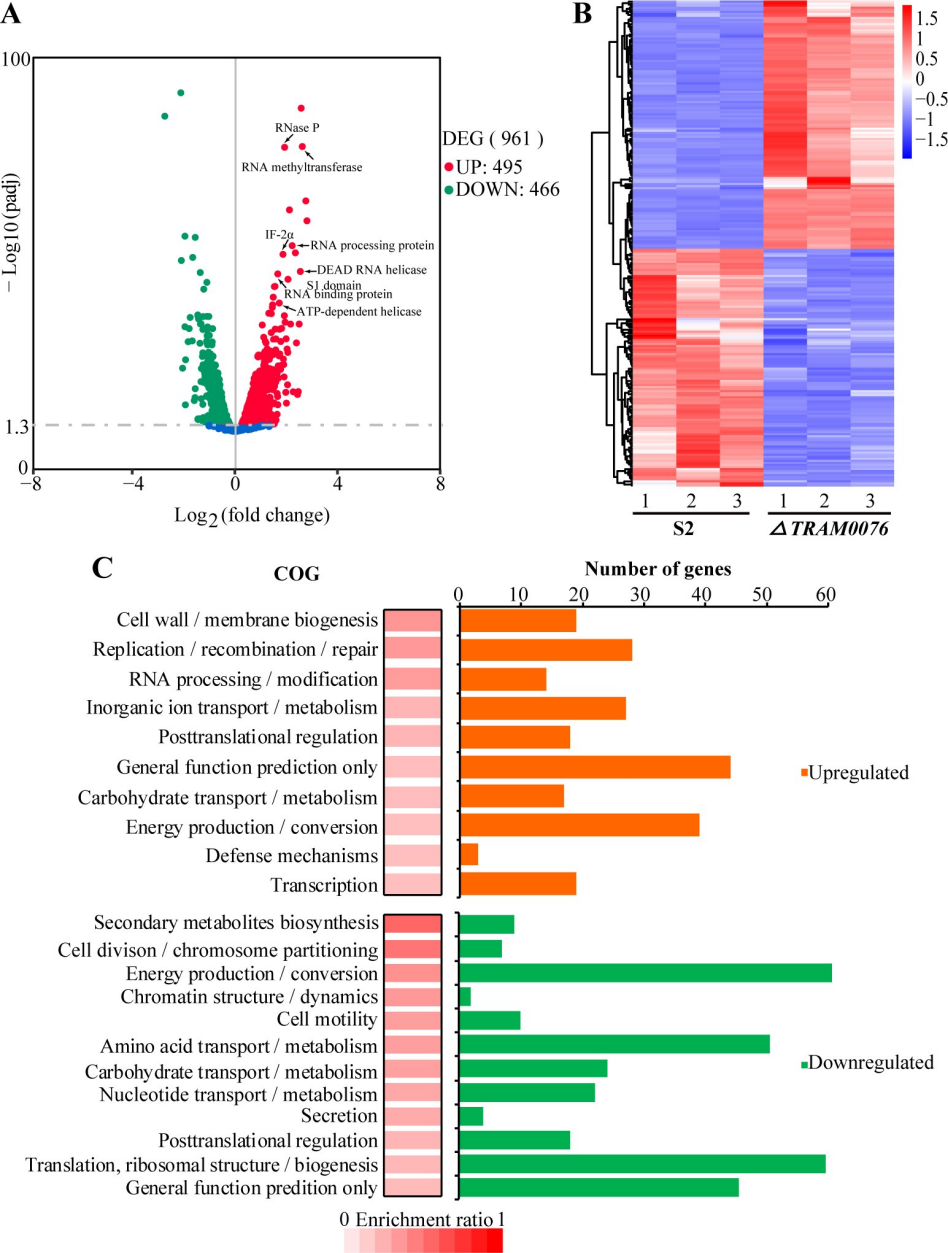
Deletion of the gene coding TRAM0076 changes the transcriptome of *Methanococcus maripaludis*. (**A**) A volcano diagram shows the distribution of 961 differentially expressed genes (DEG) in the Δ*0076* mutant. Red and green dots refer to 495 up- and 466 down-regulated genes, respectively. The dashed line denotes the thresholds for defining the differential expression by an adjusted P value Padj<0.05, which was determined by a DESeq algorithm as described in Methods. Arrows identify genes that were upregulated in the Δ*0076* mutant and encode proteins interacting RNAs. (**B**) Hierarchical clustering for the differentially-expressed genes in triplicate cultures of the Δ*0076* mutant. The heat plot representation of the log2 of differential expression fold change is shown with color intensity, by red and blue representing the maxima and minima abundance, respectively. (**C**) The functional category enrichment ratio was calculated as the gene numbers of up- or down-regulated genes divided by the total numbers of genes in each functional category, and the top 10 and 12 mostly enriched up- and down-regulated gene categories were shown respectively. The enrichment intensity (%) (red box) and the gene numbers that account for the up (orange)- or down (green)-regulated genes in the listed enriched category were also shown.

To learn more about the physiological function of TRAM0076, the transcriptome of the *MMP0076* deletion mutant was compared to that of the parental strain by RNA-seq, which was implemented with biological triplicates (S1 Dataset). As expected, the read count for the transcript of *MMP0076* ranked in top 20% of the highly expressed genes in the parental strain but was hardly detectable in the mutant, indicating a complete deletion of *MMP0076*. Importantly, transcription of the adjacent genes, *MMP0075* and *MMP0077*, were not significantly affected in the mutant, indicating that the mutation did not have polar effects on these neighboring genes. Using Padj <0.05 by the DESeq algorithm as a threshold of significant differences [30, 31], the expression of more than nine hundred genes (55% of total genes) was changed by the *MMP0076* deletion (Fig 2A and 2B, S1 Dataset), suggesting that TRAM0076 was a factor globally affecting the transcriptome. It is noteworthy that the most markedly upregulated genes in Δ*0076* included several RNA unwinding or binding proteins, such as DEAD RNA helicase (*MMP0457*), an ATP-dependent RNA-helicase (*MMP1141*), an RNA binding S1 domain protein (*MMP1127*), and translation initiation factor IF-2α (*MMP1707*) (Fig 2A), implying a possible RNA metabolism stress upon the loss of TRAM0076. Functional category analysis indicated that most of the down regulated genes in Δ*0076* were those involved in energy production and conservation, such as those for CO_2_ reduction to methane, cofactor biosynthesis, and V-type ATP synthase complex (Fig 2C, S1 Dataset). This could explain the growth reduction of the Δ*0076* mutant. Collectively, differential transcriptomic analysis indicated that TRAM0076 globally affects the transcriptome and thus suggests that it could be an important factor involved in cellular RNA metabolism.

### TRAM0076 protein exhibits RNA chaperone activity

To probe whether TRAM0076 has RNA chaperone activity similar to that of the bacterial cold shock proteins, its complementation of the deletion phenotype of cold shock proteins in *E*. *coli* was tested. *MMP0076* was cloned into the pINIII expression vector and transformed into the *E*. *coli* mutant BX04, which has a quadruple deletion of *cspA*, *B*, *G* and *E* and does not grow at low temperatures [32]. TRAM0076 complemented the cold-sensitive phenotype by recovering growth of the *E*. *coli* mutant BX04 at low temperatures (S2A Fig). PAGE of cell extracts readily demonstrated the IPTG-dependent expression of the Csp proteins and TRAM0076, whose expression was confirmed by mass spectroscopy (S2B Fig). This demonstrates that TRAM0076 shares some of the biochemical activities of Csps.

**Fig 3:**
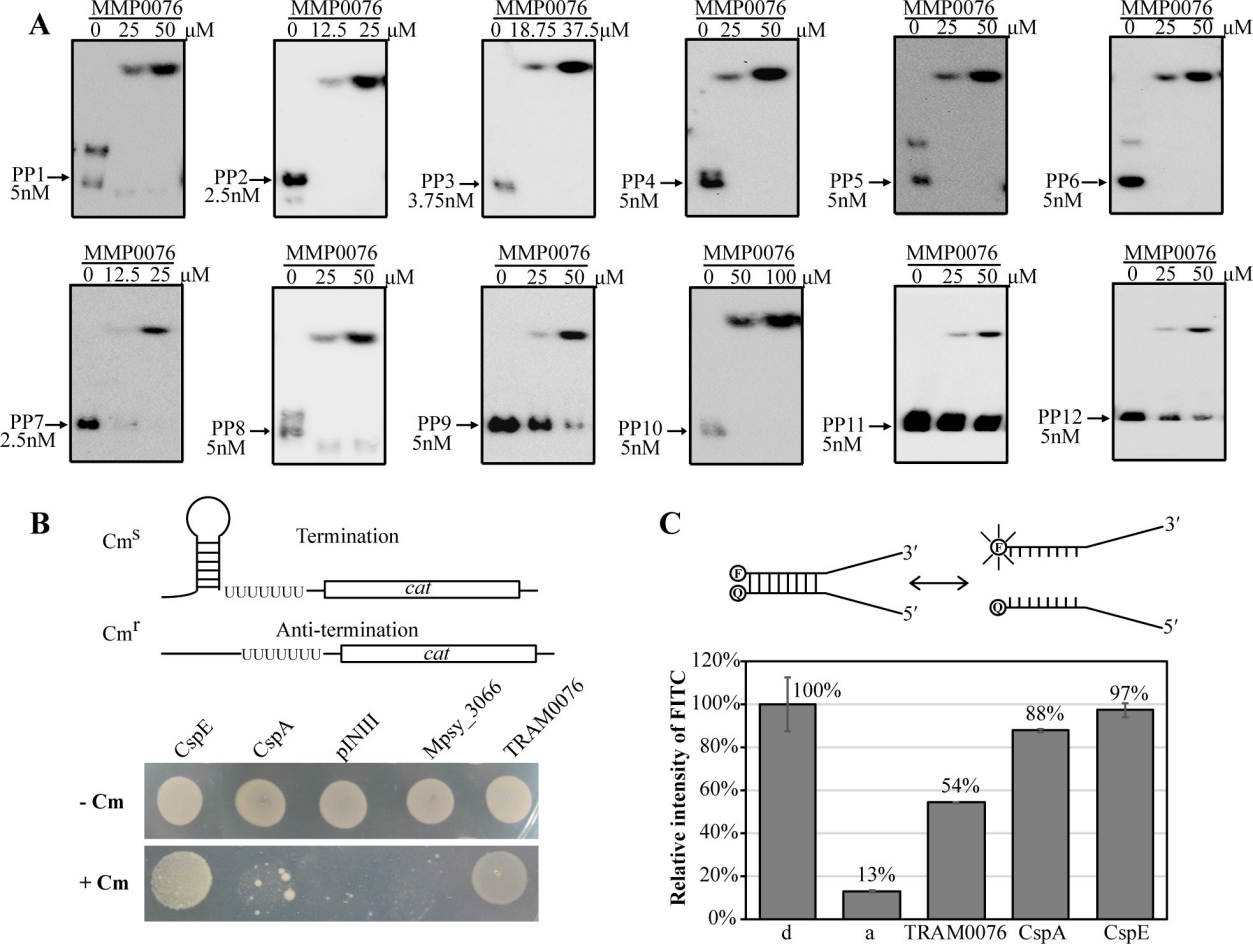
RNA chaperone activity of TRAM0076. (**A**) TRAM0076 binding to RNA pentaprobes (PP). Twelve 3′ biotinylated RNA pentaprobes (PP), PP1-PP12, were used as the substrates. Purified recombinant TRAM0076 was added to the binding reaction at indicated concentrations, and RNA-EMSA (rEMSA) was performed to determine binding. Arrows denote the free RNA probes. (**B**) Transcriptional antitermination activity of TRAM0076 determined in *E*. *coli* RL211, which carries a *cat* gene cassette positioned downstream of the *trpL* terminator (upper panel). Melting of the structured RNA of the *trpL* terminator allows expression of the chloramphenicol resistance gene *cat* and growth on plates with chloramphenicol. Strain RL211 transformed with pINIII vector alone or the pINIII carrying *MMP0076*, *cspE*, *cspA*, and *Mpsy_3066* (lower panel). Mid-exponential cultures were induced, diluted 10-fold and spotted on LB plates containing 50 μg/ml ampicillin and 1 mM IPTG, plus or minus 30 μg/ml chloramphenicol (+/-Cm). The plates were incubated at 37°C for 2–3 days. (**C**) Using a FITC fluorescence molecular beacon system (upper panel) to measure the *in vitro* nucleic-acid melting activity of TRAM0076. Unfolding of the duplex oligodeoxynucleotides relieves quenching and causes an increase in fluorescence intensity. The fluorescence of the heat-denatured molecular beacon (d) is 100%, that of the re-annealed one (a) is background. Addition of 20 μM (final concentration) of recombinant TRAM0076 protein caused the fluorescence to increase, though less than that caused by the same concentrations of the *E*. *coli* CspA and CspE proteins (lower panel).

To determine the RNA chaperone activity more directly, we first determined if TRAM0076 has RNA binding ability and if the binding is sequence specific. Twelve RNA Pentaprobes (PP) were tested as substrates in RNA electrophoretic mobility shift assay (rEMSA). These RNAs are about 100 nt in length and possess all possible 5-nt combinations (1024) [33]. Each of the 3′-end biotinylated RNA Pentaprobes was incubated with purified TRAM0076 protein, and TRAM0076-RNA complexes were found with all 12 Pentaprobes at approximate ratios of 5000 to 10000:1 (Fig 3A). The ability to bind every RNA Pentaprobe suggested that TRAM0076 binds RNA without obvious sequence specificity. To determine the RNA binding affinities of TRAM0076 more quantitatively, a surface plasmon resonance (SPR) assay was employed. The apparent K_D_ values of TRAM0076 were 4.6 and 6.3 μM for the Pentaprobes PP1 and PP10, respectively. For comparison, the K_D_ values for CspA were both about 1 μM (S3 Fig). Together, these experiments indicated that TRAM0076 binds RNA without significant sequence specificity, one of the key properties of RNA chaperones.

Next, the transcription antitermination ability of TRAM0076 in *E*. *coli* was measured. Strain RL211 of *E*. *coli* carries a *cat* (chloramphenicol resistance) gene immediately downstream of a strong *trpL* transcriptional terminator (Fig 3B upper panel) [34], which serves as an effective system in testing the transcription antitermination activity of Csps [7, 35]. In the presence of an RNA chaperone, the hairpin structure of the *trpL* RNA terminator is unwound, so the downstream *cat* is expressed, and RL211 becomes resistant to chloramphenicol. Similar to the positive control CspE from *E*. *coli*, expression of TRAM0076 led to chloramphenicol resistance (Fig 3A lower panel). In contrast, the empty expression vector pINIII and expression of TRAM3066, which binds but does not unfold RNA structure [28], did not. Thus, TRAM0076 possesses transcription antitermination activity similar to *E*. *coli* CspE, a well-established RNA chaperone.

A molecular beacon (MB) assay [35] was then performed to quantitatively test the *in vitro* duplex nucleic-acid unfolding activity of TRAM0076 protein. Two partially complementing oligodeoxyribonucleotides, one of which was FITC labeled at the 5′ terminus and the other labeled with a quencher (BHQ1) at the 3′ terminus, were used as substrates (Fig 3C). Oligodeoxyribonucleotides are commonly used in the MB assay [7, 35] as they are more resistant to the trace RNase contamination in buffer or purified recombinant proteins than oligoribonucleotides. The annealed MB yielded only 13% of the fluorescence of the denatured form due to the quenching of the FITC fluorescence by BHQ1. When the annealed MB substrates were unfolded by the *E*. *coli* CspA or CspE, the fluorescence was dramatically increased to 88% and 97% of the value of the denatured form. For comparison, addition of purified TRAM0076 (20 μM) increased the fluorescence intensity to about 54% (Fig 3C) of the denatured values. Thus, both in the *E*. *coli* cells and *in vitro*, TRAM0076 displayed the duplex nucleic-acid unfolding capability, another characteristic of RNA chaperones.

### Key residues of TRAM0076 are involved in unfolding structured RNA

**Fig 4:**
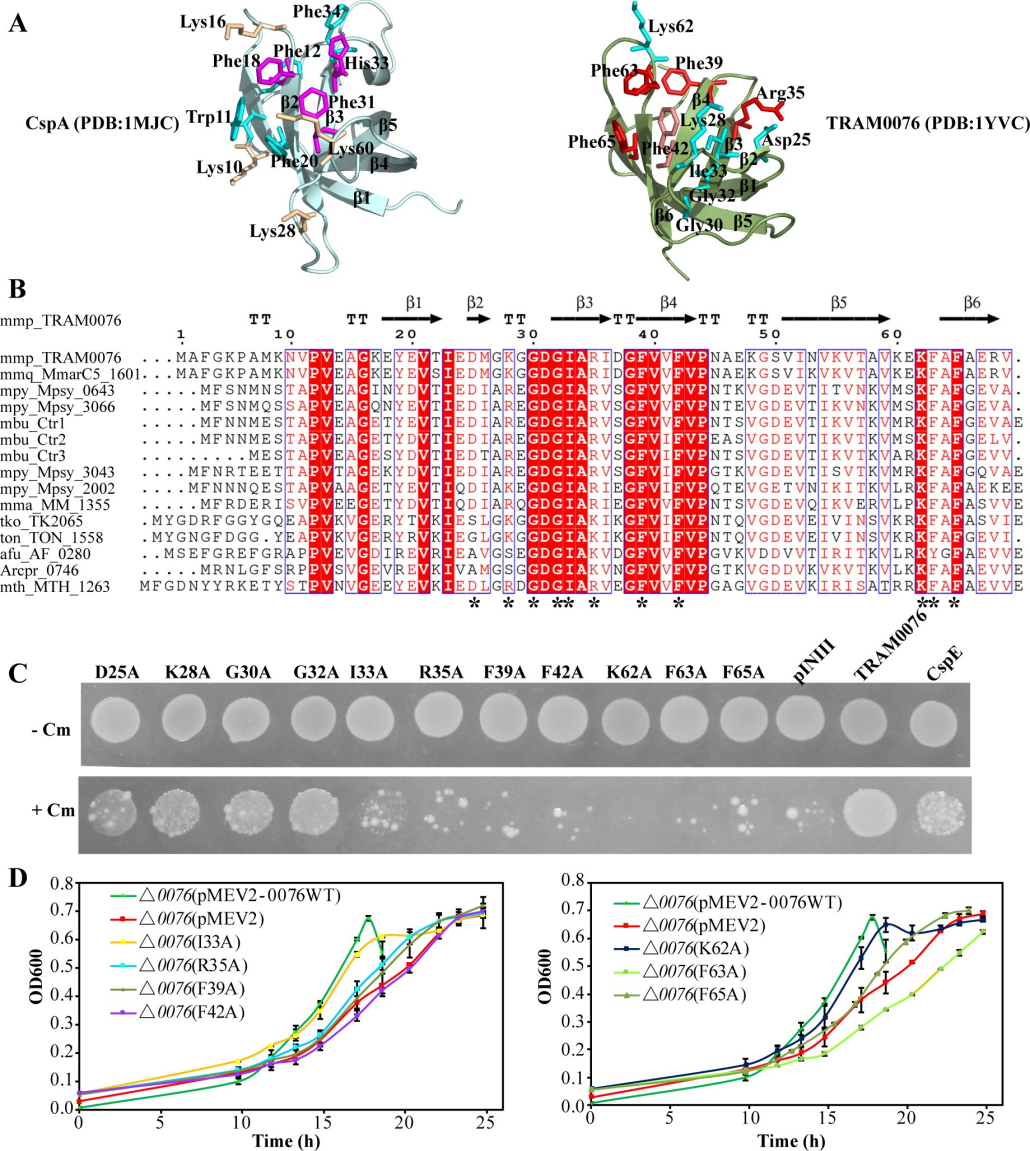
Identification of key amino acid residues of TRAM0076 required for nucleic-acid unfolding activity. (**A**) Structural comparison between *E*. *coli* CspA (left) and TRAM0076 (right) predicts the potential substrate binding surface and the key residues (sticks) in TRAM0076 equivalent to the residues essential for CspA unfolding activity. These residues are indicated by stars underneath the sequences in Fig **B**. Red sticks indicate the key residues for TRAM0076’s structured RNA unfolding activity in Fig **C** and the growth complement in Fig **D**. (**B**) Sequence alignment of TRAM proteins from some Archaea. Amino acids that are identical (red shadowed), highly conserved (framed) and conserved (orange letters) are indicated. Arrows and ′TT′ indicate the β-sheets and turns, respectively. Asterisks indicate the amino acids selected for substitutions. (**C**) Transcriptional antitermination assay of the TRAM0076 mutants in *E*. *coli* RL211. Using the same procedure as in Fig 3**B**, growth of *E*. *coli* RL211 cloned with each of the *MMP0076* mutants was determined on LB plates plus (+Cm) or minus (-Cm) chloramphenicol. (**D**) Complementation in *M*. *maripaludis* by the substitution mutants in the *MMP0076* deletion strain (Δ*0076*). Growth was measured at 37°C for triplicate cultures. Averages and standard deviations are shown.

In the *E*. *coli* Csps, aromatic and positively charged amino acid side chains play important roles in unfolding RNA structures by base stacking and electrostatic compensating interactions [3, 7]. To obtain insights into the key residues of TRAM0076 in unfolding duplex RNAs, the structure of TRAM0076 (PDB:1YVC) was first compared with that of CspA (PDB:1MJC) (Fig 4A). Although there is no sequence similarity, apparently similar OB fold structures formed, mainly by a five-stranded antiparallel β-barrel, in both TRAM0076 and CspA. The CspA RNA binding surface comprises β1 to β3. In TRAM0076, β3 to β6 was predicted to associate with RNA. Notably, structurally equivalent aromatic (Phe39, Phe42, Phe63 and Phe65) and positively charged (Lys28, Arg35 and Lys62) amino acids that are essential to the RNA chaperone activity of CspA were also found in TRAM0076. Sequence alignment of archaeal TRAM homologs indicated that these aromatic and positively charged residues were highly conserved (Fig 4B), further suggesting their importance. Thus, these seven and other four conserved residues (Asp25, Gly30, Gly32 and Ile33) within the putative RNA binding surface of TRAM0076 were selected for site-directed mutagenesis. Replacement of the four aromatic residues (Phe39 and Phe42 in β4 sheet and Phe63 and Phe65 in β6 sheet) and two positively charged residues (Arg35 in β3 sheet and Lys62 in β6 sheet) with alanine greatly reduced the structured RNA unfolding activity of TRAM0076 in the transcription antitermination assay in *E*. *coli* RL211 (Fig 4C). Although the Ile33 mutation also failed to suppress transcription termination in this assay, Western blotting of cell extracts detected an obvious reduction of TRAM0076 protein for this mutation, indicating that the mutant protein was labile.

### The structured RNA unfolding activity is necessary for the physiological function of TRAM0076

If structured RNA unfolding activity was necessary for the physiological activity of TRAM0076, mutations that inactivate the activity should be unable to complement the growth defect of the Δ*0076* mutant. To test this hypothesis, the eleven single-site mutations were each transformed into the Δ*0076* strain of *M*. *maripaludis*. Five TRAM0076 mutants that failed to suppress transcription termination in *E*. *coli*, R35A, F39A, F42A, F63A and F65A, also did not complement the *MMP0076* deletion (Fig 4D). The mutant K62A partially complemented. In contrast, the mutants with transcription antitermination activity, K28A, G30A, and G32A, as well as I33A fully restored growth to the deletion mutant (Fig 4D and S4 Fig). Together, these results indicate that amino acid residues key for structured RNA unfolding and transcription antitermination activity in RL211 were also important for TRAM0076’s physiological role in *M*. *maripaludis*.

### Transcription start site mapping predicts 5′UTR structures in the methanococcal transcripts

Structural signals such as hairpins of the mRNA 5′UTRs are common elements interacting with RNA binding proteins [5, 20, 36, 37]. To identify potential interactions between TRAM0076 and methanococcal 5′UTRs, the genome-wide transcription start sites (TSSs) and 5′UTRs were mapped in *M*. *maripaludis* by dRNA-seq as previously described [20]. In summary, 461 gTSSs were found for 325 monocistronic and 136 polycistronic operons comprising a total of 720 genes. Of these, 249 operons comprising 54% of genes possess 5′UTRs in length of 20–60 nt, or long enough to form secondary structures (S2 Dataset). Notably, 155 of the 466 down-regulated transcripts in the Δ*0076* mutant possessed 5′UTRs, and 65% (101 mRNAs) of which were 20–60 nt in length (S5 Fig). As predicted by Mfold software (unafold.rna.albany.edu/?q=mfold), transcription terminator-like hairpin structures were common in the 5′UTRs of this length (S6 Fig). These structures in the 5′UTRs of the down-regulated transcripts are candidate targets of TRAM0076 antiterminator activity (see below). In addition, 38 of the 104 up-regulated transcripts possessed 20–60 nt 5′UTRs. Moreover, a few of the genes upregulated in the Δ*0076* mutant possessed much longer 5′UTRs, such as 223 nt in the DEAD RNA helicase (*MMP0457*) transcript, 116 nt in the F_420_-non-reducing hydrogenase subunit (*vhcD*, *MMP0821*) transcript and 104 nt in a peptide methionine sulfoxide reductase (*MMP084*8) transcript (S2 Dataset). These secondary structures are additional targets for TRAM0076. Because expression of these genes is upregulated in the Δ*0076* mutant, antitermination is not a likely mode of action.

### TRAM0076 unfolds 5′UTR structures and facilitates transcription of some down-regulated genes

**Fig 5:**
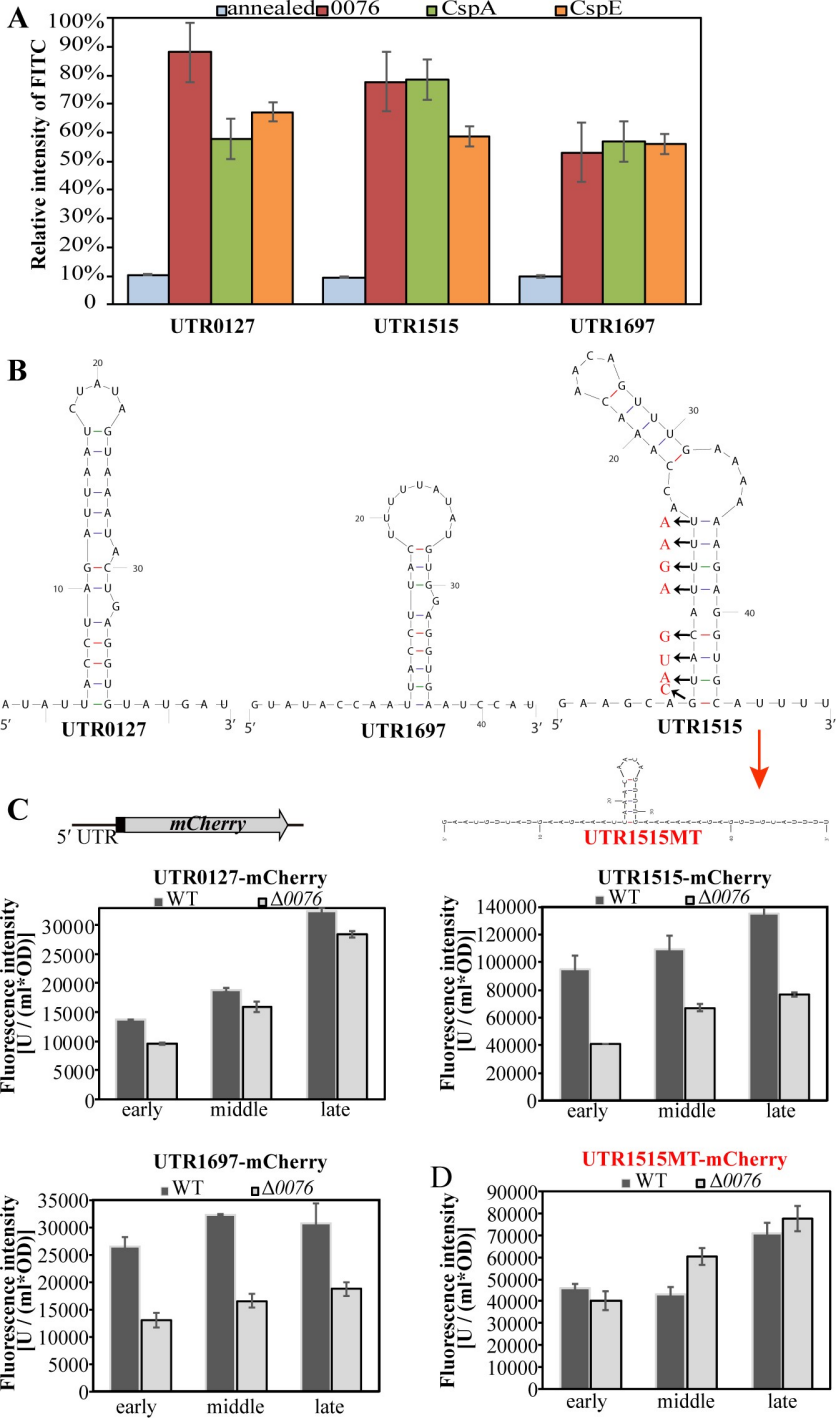
TRAM0076 promotes gene expression by unfolding 5′UTR structures. (**A**). Molecular beacons of the 5′UTR oligodeoxynucleotide fragments from *MMP0127* (UTR0127), *MMP1515* (UTR1515) and *MMP1697* (UTR1697) were constructed by FITC and BHQ1 labeling at the 5′ and 3′ termini, respectively. Unfolding by TRAM0076 and *E*. *coli* CspA and CspE were determined using the same procedure as in Fig 2C. (**B**). RNA structures in the 5′UTRs were predicted by Mfold. The arrows specified bases substituted in the 5′UTR of *MMP1515* to abolish the hairpin structure and to construct UTR1515MT. (**C**). Each of the 5′UTRs from *MMP0127*, *MMP1515* and *MMP1697* was fused upstream of the mCherry ORF to construct a 5′UTR-mCherry reporter system (upper) and then transformed into the Δ*0076* mutant and parental S0001 strain of *M*. *maripaludis*. At the early-, middle- and late-exponential growth phases, fluorescence at 575 nm for excitation and 610 nm for emission were assayed for the parental strain (WT) and the Δ*0076* mutant (Δ*0076*). Fluorescence intensities of mCherry were assayed in triplicate cultures, and average and standard deviations are shown. (**D**). UTR1515MT-mCherry reporter was constructed by a fusion of the UTR1515MT upstream the mCherry ORF and transformed into the wild-type strain and the Δ*0076* mutant, and fluorescence intensities of mCherry were assayed as for the UTR1515-mCherry reporter in Fig **C**.

To test if TRAM0076 facilitates transcription via unfolding 5′UTR hairpin structures in *Methanococcus*, the 5′UTR leaders of three transcripts (*MMP0127*, *MMP1515* and *MMP1697*) whose abundances were reduced in the Δ*0076* mutant and possessed potential hairpin structures were examined in the molecular beacon system (Fig 5A). Mfold predicted hairpin structures of 9–13 Watson-Crick base pairs in the three 5′UTRs (Fig 5B). The three 5′UTR leaders labeled with FITC fluorophore at the 5′ terminus and quencher (BHQ1) at the 3′ terminus were used as MB substrates. Addition of TRAM0076 protein (20 μM) to the three MB substrates increased the relative FITC fluorescence intensity to 55–88%, confirming the ability of TRAM0076 to unfold the predicted hairpins in these 5′UTRs, which could be the basis for regulation through transcription antitermination.

To determine if TRAM0076 actually facilitates transcription of its targeted mRNA through unfolding the 5′UTRs *in vivo*, a mCherry reporter was fused to each of the three 5′UTRs (*MMP0127*, *MMP1515* and *MMP1697*) in the replicative vector and transformed into the Δ*0076* and parental strains of *M*. *maripaludis*. Expression of the mCherry reporter gene was assayed by measuring the fluorescence intensity of whole cells during growth. Compared to the parental strain, the expression of mCherry in the Δ*0076* mutant was reduced by approximately 50% for *MMP1515* and *MMP1697* (Fig 5C) and was slightly reduced for *MMP0127*. Furthermore, when the 5′UTR hairpin structure in *MMP1515* was abolished by base substitutions (Fig 5B, UTR1515MT), the differential expression of mCherry reporter in the Δ*0076* and parental strains was lost (Fig 5D). These results provided direct evidence that TRAM0076 can facilitate transcription of at least some of its targets via its RNA chaperone activity acting on 5′UTR structures.

Given that cold shock induced TRAM0076 expression by 50–80% (Fig 1D), expression of mCherry reporter was also tested in cold shocked cells. As anticipated, the fluorescence of cells with mCherry fused to each of the three 5′UTRs increased about 1.4-fold in the cold shocked parental strain but not the cold shocked Δ*0076* mutant. Cold shock induction was also not observed in the mCherry reporter of UTR1515MT, the hairpin structure of which was abolished by nucleotide substitutions (S7 Fig). This experiment indicated that cold induced TRAM0076 increased the transcription of its targeted transcripts, further verifying the ability of TRAM0076 in facilitating transcription for some of its targets with 5′UTR hairpin structures.

### RNA co-immunoprecipitation determines the *in vivo* binding of TRAM0076 to RNAs

**Fig 6:**
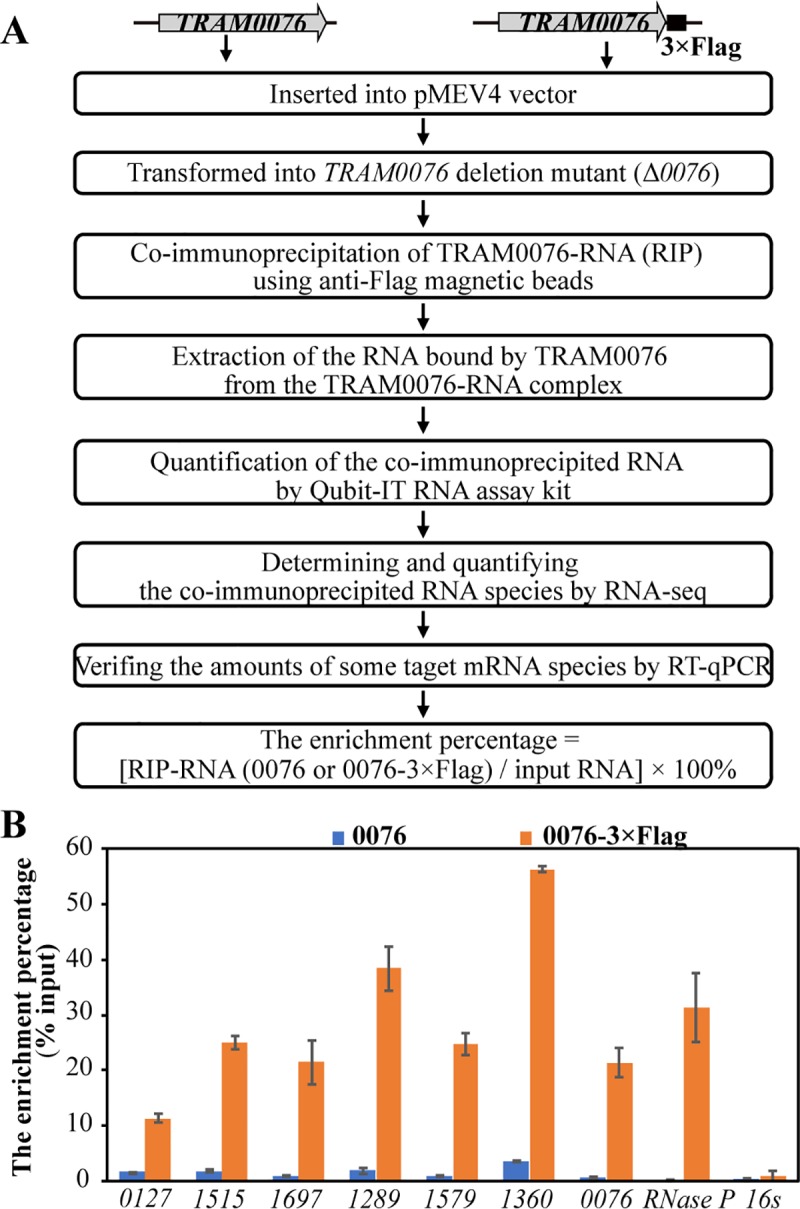
Determination of Flag-tagged TRAM0076 co-immunoprecipitated RNAs. **(A**) Flowchart of RNA immunoprecipitation experiment. (**B**) qPCR quantification of selected transcripts precipitated by Flag- (0076–3×Flag) and un-tagged (0076) TRAM0076. Using the precipitated RNAs as templates, qPCR determined the copies of the 5′ fragments using the primer pairs in the S2 Table. The enrichment percentages by Flag- and un-tagged TRAM0076 were calculated by the method indicated in (**A**). Total RNAs were collected from three batches of cultures, and the average of percent precipitated and standard deviations are shown.

To explore more possible action mechanisms of TRAM0076, the targets of TRAM0076 in the *M*. *maripaludis* cells were determined by means of RNA co-immunoprecipitation (RIP) (Fig 6A). The Flag-tagged *MMP0076* gene was expressed in the Δ*0076* mutant using the replicative vector pMEV4, and the untagged *MMP0076* complementation strain served as a mock control. TRAM0076 bound RNAs collected from Flag-tagged and untagged TRAM0076 complemented strains were co-immunoprecipitated using the anti-Flag magnetic beads. The corresponding RNA pools prior to RIP (the Input RNAs) were also examined. Sensitivity to ribonucleases confirmed that the RIP collected nucleic acid was RNA. The RNA abundance in the Flag-tagged TRAM0076 precipitant was much higher than that of the mock control (S8A Fig). Compared to the Input RNA pool, more mRNAs were precipitated than rRNAs (S8B Fig). Flag-tagged TRAM0076 precipitated RNAs were subsequently sequenced and both the readcount and relative abundance calculated as Fragments Per Kilobase of transcript per Million fragments mapped (FPKM) were listed in S3 Dataset. The amount of RNA precipitated in the untagged TRAM0076 mock control was too low to be sequenced. By taking the FPKM median value of 320 (S9 Fig) as a threshold of RIP collected RNAs, Flag-tagged TRAM0076 precipitated at least 900 transcripts. This result suggests that TRAM0076 binds abundant cellular RNAs *in vivo*, a trait consistent with the low sequence specificity measured *in vitro* and the characteristic of other RNA chaperones.

Among the RNAs precipitated by Flag-tagged TRAM0076, some were highly enriched. These included the messages encoding RNase P protein (*MMP1407*) and its RNA component, two histones (*MMP0386* and *MMP1347*), translation initiation factors (*MMP1406*), elongation factor 1α and β subunits (*MMP1370* and *MMP1401*), RNA polymerase subunits RpoN and RpoF (*MMP1326* and *MMP0092*), ribosomal proteins (rpl40e, *MMP0151*; rps27e, *MMP1708*; and rplX, *MMP0060*), some methanogenesis genes (*mcrG*, *MMP1558*; *mtrG*, *MMP1566*), and an ArsR family transcriptional regulator (*MMP1442*). Of note, the TRAM0076 transcript was also highly enriched, suggesting a potential self-regulation of its own expression. In contrast, some RNAs were conspicuously unenriched, including rRNAs and tRNAs. These stable RNAs are either highly structured or tightly associated with proteins.

Precipitation of some of the highly enriched RNAs and downregulated methanogenic genes in the Δ*0076* mutant were selected for validation by quantitative RT-PCR (qPCR; Fig 6B). While the 16S rRNA was not enriched in the Flag-tagged TRAM precipitant, the transcripts for TRAM0076, RNase P RNA component, two ribosomal subunits (*MMP1289* and *MMP1579*), a RNAP subunit (*MMP1360*), and three downregulated transcripts (*MMP0127*, *MMP1515* and *MMP1697*) were enriched in the Flag-tagged TRAM0076 precipitant by 6.8 to 100-fold higher than in the mock precipitant. These experiments confirmed that TRAM0076 bound a large number of transcripts *in vivo*.

## Discussion

RNA chaperones, through RNA binding and structured RNA unfolding activities, ensure the correct folding of targeted RNAs and enable them to adopt their functionally active states, thereby playing important roles in control of gene expression [3, 38, 39]. However, compared to bacterial and eukaryotic RNA chaperones, little is known about archaeal RNA chaperones, particularly of their biological significance and functional mechanisms. This work, through physiological, genetic and transcriptomic, and molecular and biochemical studies, reports for the first time that an archaeal RNA chaperone TRAM shapes the transcriptome and ensures the optimal growth of an archaeon.

The methanococcal TRAM0076 displays the defining properties of an RNA chaperone. It binds RNAs without obvious sequence specificity both *in vivo* and *in vitro*, and unfolds RNA hairpins and other secondary structures. Importantly, mutations of the amino acid residues essential for RNA unfolding also eliminate the biological effects of TRAM0076 in *Methanococcus*, demonstrating that its biological role depends on its structured RNA unfolding activity. Further, the methanococcal TRAM0076 facilitated transcription of some genes by unfolding the 5′UTR structures, presumably to prevent premature transcriptional termination or pausing.

Although TRAM0076 shares the RNA chaperone activities of the bacterial cold shock proteins, unlike the *E*. *coli* Csps, which exert roles mainly when the organism encounters cold shock or lower temperatures [4], TRAM0076 appears to play a more fundamental role in *Methanococcus* as deletion of the gene impaired the growth at both optimal and lower temperatures. Accordingly, absence of TRAM0076 causes changes in the expression of 55% of the total genes, much more than the gene numbers that are affected by Csps [40]. Thus, this archaeal RNA chaperone affects transcription globally. Meanwhile, the prevalence of 5′UTRs in methanococcal transcripts could provide the wildly distributed targets for TRAM to implement a regulatory role on transcription. For instance, 65% of the down-regulated transcripts in the Δ*0076* mutant possess a 5′UTR of 20–60 nt, and both *in vivo* and *in vitro* experiments demonstrate that TRAM0076 unfolds 5′UTR structures. Therefore, one of the mechanisms that TRAM0076 facilitates archaeal transcription would be prevention of 5′UTR structure formation by unfolding the kinetically trapped stem-loop structures and transcription antitermination activity, similar to that of the bacterial Csp proteins [5, 37].

In addition to unfolding 5′UTR structures, TRAM0076 may exert roles in mediating gene expressions via other RNA chaperone activities, such as binding the RNAs so to prevent the formation of the kinetically trapped non-active conformations. Deletion of *MMP0076* also causes increased expression of about one third of the total genes, including a DEAD/DEAH RNA helicase (*MMP0457*) and a SAM-dependent methyltransferase (*MMP0874*). Transcripts of the two genes were precipitated by the Flag-tagged TRAM0076, evidence that they were bound by TRAM0076 *in vivo*. Moreover, the transcripts of *MMP0457* and *MMP0874* possess long 5′UTRs of 223 nt and 84 nt, respectively. As the two genes are upregulated in the Δ*0076* mutant, TRAM0076 must act on them by some mechanisms other than transcriptional antitermination. TRAM0076 also bound the transcript of an AsrR family transcription regulator. By controlling the expression of other regulatory proteins, TRAM0076 could exert an indirect role in regulation of gene expression. It is worthy to note that, unlike the *in vitro* experiment results of the cold-responsive TRAM protein Crt3 from *M*. *burtonii* [27], TRAM0076, at the physiological level, did not show a preferential binding to tRNA and rRNAs in the *in vivo* assay, though the two proteins have highly similar sequences (Fig 4B). While the apparent difference in RNA targets determined for the two homologous TRAM proteins could have resulted from the different experimental approaches, it is also possible that small differences in the protein sequences significantly changed the substrate specificities.

TRAM0076 is a very small protein with only a single TRAM domain. Because it lacks a regulatory domain, its physiological activity is probably controlled via its expression. Given that TRAM0076 binds its own transcript, an auto-regulation mode for its expression is also predicted. Expression of TRAM0076 increases following cold shock. For the bacterial CspA, temperature-dependent regulation results from different structures adopted by its 5′UTR at different temperatures [41]. However, the mechanism of cold shock induction of TRAM0076 must differ because the structure of its 5′UTR at 37°C and 4°C are predicted to be the same. In addition to cold shock, the abundance of TRAM0076 also increases during the stationary growth phase. However, little is known about growth phase regulation in the archaea.

TRAM0076 is estimated to be 0.05–0.1% of the cellular proteins at the exponential growth phase. Given a protein content of 4.3 × 10^−13^ g per cell [42] and a molecular mass of 7.4 KDa for TRAM0076, each *M*. *maripaludis* cell is estimated to contain ~17,500 to 35,000 molecules of TRAM0076. Given the aqueous volume of 10 × 10^−16^ L per cell [43], the calculated molar concentration of TRAM0076 is 29 to 58 μM. Given its relative high affinity of ~5 μM for Pentaprobe RNAs (S3 Fig) and low *in vivo* binding of the highly structured tRNAs and rRNA, it is reasonable to expect that most of the cellular mRNAs will be bound to TRAM0076 under physiological conditions. By binding renascent mRNAs and remodeling the conformations to facilitate the transcription and / or translation via its RNA chaperone activity, TRAM0076 could also associate with transcription and / or translation machineries that are involved in the fundamental processes of life. Thus, abolishment of these associations in the Δ*0076* deletion mutant could impair translation and transcription and hence growth on multiple levels.

The TRAM domain occurs in the N- or C-terminal regions of proteins with diverse functions, such as RNA methylases, translation initiator factor eIF2β, ribosomal protein S2 and methylthiolase, which are widely distributed in all organisms including Archaea. Therefore, Anantharaman *et al*. proposed that TRAM domains are mobile and fused to diverse proteins during evolution [26]. The observation that the stand-alone TRAM domain proteins are widely distributed in Archaea and plays a fundamental role in mRNA expression and the growth of modern Archaea further suggests that RNA chaperones may also have been important elements in the ancestor to the archaeal lineage.

Moreover, RNA chaperones are also involved in biological processes in addition to cold protection of organisms. When expressed in *Arabidopsis*, rice and maize, the two bacterial RNA chaperones, the *E*. *coli* CspA and the *Bacillus* CspB, improve growth of the plants under water-limited conditions and other abiotic stresses [44]. Similarly, ectopic expression of two TRAM proteins from *Methanolobus psychrophilus* R15 significantly improves the tolerance of the transgenic rice to drought and high salinity stresses [45]. Given that the archaeal TRAM can replace the function of the *E*. *coli* cold shock proteins, this ancient RNA chaperone may function in a number of basic cellular processes regardless of the types of organism.

## Materials and methods

### Microbial strains and culture conditions

The microbial strains used in this study and their characteristics are listed in S1 Table. *Escherichia coli* strains were routinely grown at 37°C in Luria-Bertani (LB) medium with shaking.

*M*. *maripaludis* strains were cultured in pre-reduced DSMZ medium 141 containing 40 mM formate at 37°C under a gas phase of N_2_/CO_2_ (80: 20, v/v; 0.1 MPa) as previously described [46, 47]. Growth was determined by measurement of the optical density of at 600 nm (OD600).

### Construction of *MMP0076* deletion mutant and ectopic complementation

Plasmids used for genetic manipulations are listed in S1 Table. The *MMP0076* gene of *M*. *maripaludis* S0001 was deleted by replacement with the *pac* cassette, which encodes puromycin resistance, through homologous recombination using the integration vector pIJA03 and polyethylene glycol (PEG)-mediated transformation [47, 48]. For complementation analysis, the coding region of *MMP0076* was PCR amplified and inserted into the shuttle vector pMEV2 at the NsiI/XbaI site to generate pMEV2-0076. After verifying the sequence, pMEV2-0076 was transformed into the Δ*0076* mutant to construct *MMP0076-com* strain, and the transformants were screened in the presence of 1 mg/ml neomycin. As a control, the empty vector pMEV2 was transformed into both the wild-type strain and the Δ*0076* mutant, respectively.

### PCR amplification, cloning, and mutagenesis

For expressing TRAM0076 in *E*. *coli* BX04 and RL211 strains, the open reading frame of *MMP0076* was PCR amplified from the genomic DNA using primer pairs P3/P4 (S2 Table) and then cloned into the NdeI/BamHI sites of pINIII (pIN-III-lppP-5), resulting in the expression plasmid pIN-0076. In parallel, the *E*. *coli cspA* and *cspE* and *Mpsy_3066* (encoding TRAM3066) of *M*. *psychrophilus* R15 [28] were cloned as controls.

For overexpression of the His_6_-tagged recombinant TRAM0076, *MMP0076* sequence was amplified by primer pairs P11/P12 (S2 Table) and cloned into NcoI/XhoI sites of pET28a (Novagen, Madison, USA), resulting in p28a-0076. Similarly, the *E*. *coli cspA*, *cspE* and *M*. *psychrophilus Mpsy_3066* were also cloned into pET28a, resulting in the expression plasmids as p28a-cspA, p28a-cspE and p28a-3066 using the primer pairs listed in S2 Table.

To introduce the site-directed mutations in TRAM0076, the replicative plasmids pIN-0076 and pMEV2-0076 served as templates for expression in *E*. *coli* RL211 [34] and *M*. *maripaludis* [48], respectively.

### Overexpression and purification of proteins

To produce recombinant TRAMs, plasmids p28a-3066, p28a-0076 and its mutants, p28a-cspA and p28a-cspE were each transformed to *E*. *coli* BL21(DE3)plysS. Expression of the 6×His tagged proteins were induced by IPTG (isopropyl-D-thiogalactopyranoside) and purified as described previously [28] by Ni^2+^-nitrilotriacetatic acid-agarose columns (Novagen), HiTrap Q HP anion exchange columns and gel filtration chromatography according to the manufacturer’s protocols (GE Healthcare).

### RNA electrophoretic mobility shift assay (rEMSA)

Using the approach of Zhang et al. [28], rEMSA was performed for purified TRAM0076 protein. The 3′ end biotinylated Pentaprobes (PP) library RNAs [33] were used as substrates, and the RNA binding assay was carried out as described previously [28] with some modifications. Briefly, the 20 μl binding reaction including purified TRAM0076 protein and 3′ labeled PP RNAs was incubated at 25°C for 20 min and then electrophoresed under 100 V for 1 h in 0.5× TBE running buffer (1 mM EDTA, 45 mM Trisboric acid, pH 8.0). Free RNA and RNA-protein complexes were transferred to a nylon membrane, and after cross-linking by UV they were detected using a Chemiluminescent Nucleic Acid Detection Module kit (Thermo Scientific) according to the manufacturer’s instruction.

### Transcription anti-termination assay in *E*. *coli* and in *M*. *maripaludis*

To determine the nucleic-acid unfolding activity of TRAM0076 and its mutants, plasmids pIN-0076 and its derivatives were each transformed into *E*. *coli* RL211. The transformants were cultured overnight in LB complemented with 100 μg/ml ampicillin and then diluted a 100-fold into the fresh medium. When the culture grew to an OD600 nm of 0.4–0.6, 1 mM IPTG was added, and cultivation continued for another 2 h. Next, six μl of 10-fold diluted cultures were spotted on LB plates containing 100 μg/ml ampicillin, 1 mM IPTG, with or without 30 μg/ml chloramphenicol, and grown for 2–3 days.

To detect the role of TRAM0076 to facilitate transcription in *M*. *maripaludis*, another replicative vector pMEV4-mCherry-neo carrying a *neo* cassette (neomycin resistant) and a mCherry reporter gene was used. Each of the three 5′UTRs (*MMP0127*, *MMP1515* and *MMP1697*) was inserted upstream the mCherry gene in the vector, resulting plasmids pMEV4-0127PUO, pMEV4-1515PUO, and pMEV4-1697PUO, and then transformed into the Δ*0076* mutant and the wild-type strain, respectively. The pMEV4-1515PUOMT was constructed by the site-directed mutation of the pMEV4-1515PUO described above. Expression of the mCherry reporter gene was assayed by detecting the fluorescence at 575 nm (excitation) and 610 nm (emission) for the cultures during growth.

### *In vitro* nucleic-acid unfolding assay

A fluorescent molecular beacon system developed by Nakaminami *et al*. [35] was used to determine the nucleic-acid unfolding activity of TRAM0076. Briefly, two partially complementary oligodeoxynucleotides were labeled with FITC and BHQ1, respectively (Sangon, Shanghai, China). Similarly, FITC and BHQ1 were used to label three oligodeoxynucleotides of the 5′UTRs of *MMP0127*, *MMP1515* and *MMP1697* (Fig 4A) at the 5′ and 3′ terminus, respectively. FITC and BHQ1-labelled oligodeoxynucleotides were mixed at a ratio of 1:1, denatured at 95°C for 5 min and cooled gradually to 4°C. 20 picomole annealed oligonucleotides was incubated with 20 μM (final concentration) purified recombinant TRAM0076 at room temperature in 200 μl of 200 mM Tris-Cl (pH 7.5) and 10 mM MgCl_2._ 20 μM (final concentration) CspA and CspE were included as positive controls. Fluorescence was measured in 96-well plates using a Synergy H4 hybrid Reader (BioTek, USA) at 460 nm for excitation and 515 nm for emission.

### Comparative transcriptomics and genome-wide transcription start site mapping

Cultures at the mid-exponential phase of *M*. *maripaludis* S0001 and the Δ*0076* mutant were harvested from three independent cultures. The total RNA was extracted using TRIzol (Ambion), and the whole-transcript cDNA libraries, high-throughput sequencing and quality control (QC) were performed as previously described by Li *et al*. [20]. Uniquely mapped reads were aligned to the *M*. *maripaludis* S2 reference genome (Methanococcus_maripaludis/GCF_000011585). The differential expression analysis of transcripts between wild-type and Δ*0076* mutant was performed by using the DESeq algorithm with the normalized mapped read counts, and a threshold of the adjusted P value Padj <0.05 was considered as a significant differential expression [30, 31]. The Pearson correlation (R^2^) among the three biological repeats ranged 0.889 to 0.989.

Genome-wide transcription start sites (TSS) were mapped using the differential RNA-sequencing (dRNA-seq) approach as described previously [20]. After TSS calling, the operon composition and 5′ untranslated regions (UTR) were analyzed for *M*. *maripaludis* S0001 as described in [20].

### RNA co-immunoprecipitant sequencing and qRT-PCR

*MMP0076* gene tagged with a C-terminal his_6_ or a his_6_ plus 3×Flag-tag was inserted into pMEV4 that carries a *neo* cassette by using the Gibson assembly method [49] and transformed into the Δ*0076* mutant to construct MMP0076-his_6_ and MMP0076-his_6_-3×Flag strains. Cells at the late exponential phase were collected, and the pellet was washed with 1 mL of buffer A (50 mM Tris-HCl (pH7.5), 500 mM NaCl, 10% (w/v) glycerol), snap-frozen in liquid nitrogen, and stored at -80°C. RNA co-immunoprecipitation (RIP) was then performed as described previously [39, 50, 51] with some modifications. Briefly, cells were broken in the lysis buffer B (50 mM Tris-HCl (pH7.5), 1mM MgCl_2_, 150 mM NaCl, 0.5% Triton X-100 supplemented with cOmplete mini protease inhibitor (Roche) and 10 U/ml RNasin (Promega) by repeated pipetting. Prior to immunoprecipitation, 1% of the supernatant was used to prepare total RNA as input. The remainder was pre-cleared by mouse IgG-agarose (Sigma), and then incubated with 40 μl of Anti-Flag M2 Magnetic Beads (Sigma) at 4°C and pre-blocked by incubation in 1% BSA. The Co-IP anti-Flag beads were washed three times with 1 ml buffer B, and two times with 1 ml buffer B plus 150mM NaCl. RNA-bound to the Flag-tagged TRAM0076 was eluted using the FLAG Peptide (Sigma), and RNA was extracted and precipitated with ethanol with NaOAc and glycogen as carrier. Extracted RNAs were quantified by Quant-iT RiboGreen RNA kit (invitrogen).

The immunoprecipitated RNAs were treated with DNase I, and cDNA libraries were constructed using NEBNextH UltraTM Directional RNA Library Prep Kit for IlluminaH (New England Biolabs) as described previously [20]. Sequencing was performed on an Illumina HiSeq 2000 platform, and the filtrated sequencing reads were mapped to the *M*. *maripaludis* S2 genome (*Methanococcus_maripaludis*/ GCF_000011585). Read counts were normalized to the Fragment Per Kilobase of transcript per Million fragments mapped (FPKM).

Quantitative RT-PCR (qPCR) was performed to verify RIP-seq data. cDNAs were generated from 2 μg of input RNA and 10 μl of immunoprecipitated RNA by RT-PCR with random primers using Moloney murine leukemia virus reverse transcriptase (Promega). qPCR amplification was performed using the corresponding primers (S2 Table) at Mastercycler EP realplex2 (Eppendorf, Germany). To estimate copy numbers of tested mRNAs, standard curves of the corresponding genes were generated using 10-fold serially diluted PCR product as templates. RIP enriched percentage of each mRNA was calculated as the copies in RIP divided by those in the input sample times 100. 16S rRNA gene was used as a control.

### TRAM0076 complementing the cold sensitivity of *E*. *coli* BX04

Using the same approach as described previously [28], TRAM0076 complementation of the cold sensitivity of *E*. *coli* BX04 [33] was performed. Briefly, plasmid pIN-0076 was transformed into *E*. *coli* BX04 and 10-fold serial dilutions of the transformant were spotted on LB plates containing 100 μg/ml ampicillin and incubated for 2–5 days at 37°C, 22°C and 18°C.

## Supporting information

S1 Fig Phylogenetic tree of the archaeal TRAM proteins. The tree was constructed using MEGA7.0, and the bar represents 10% sequence difference. The archaeal species and their encoded TRAM proteins are indicated, and numbers following each species are the TRAM genes copies.
(TIF)

S2 Fig TRAM0076 complements the cold sensitivity of *E*. *coli* BX04. *E*. *coli* BX04 carries a deletion of the *cspABGE* operon, and its growth at low temperature is retarded. (A) Overnight liquid cultures of the *E*. *coli* strains that overexpress *MMP0076*, *Mpsy_3066*, *cspA* or *cspE* were diluted to OD600 of 0.9 with the fresh medium and then 10-fold serially diluted to 10^−5^ and spotted on LB plates supplemented with ampicillin. The plates were incubated at 18°C, 22°C and 37°C for 2-5 days with or without IPTG, as indicated. pINIII indicates the cultures carrying the empty plasmid vector. + and–, cultured in the presence or absence of IPTG, respectively. pINIII, strain carrying the empty plasmid pINIII. (B) SDS-PAGE confirmation of TRAM protein expression in strain BX04. Left: PAGE of 2.4 μg of purified CspA, CspE, TRAM3066 and TRAM0076. Right: Cell extracts from strain BX04 overexpressing cspA, cspE, Mpsy_3066 or MMP0076 were electrophoresed on 5% SDS-PAGE. + and–, cultured in the presence or absence of IPTG, respectively. pINIII, strain carrying the empty plasmid pINIII. Asterisks indicate the predicted migration of the overexpressed proteins.
(TIF)

S3 Fig Surface plasmon resonance (SPR) assay of TRAM0076 binding to RNAs. Two RNA Pentaprobes were used as substrates and immobilized to the SPR chip, and the RNA binding affinity was tested by addition of the indicated concentrations of TRAM0076. *E*. *coli* CspA and TRAM3066 are included as references. Calculated the equilibrium dissociation constant K_D_ values are shown for each pair of protein-RNAs. The protein concentrations for each reaction are color coded and labeled in the bottom row. The x axis indicates the reaction time in seconds, and the y axis shows arbitrary resonance units (RU), where 1000 RU corresponds to a surface density of 1 ng/mm^2^.
(TIF)

S4 Fig Growth curves of complementation of the substitution mutants K28A, G30A, and G32A. Complementation of the wild-type *MMP0076* (PMEV2-0076WT) was included as the reference. Growth was measured at 37°C for triplicate cultures. Averages and standard deviations are shown.
(TIF)

S5 Fig Box-plot shows the 5′UTR length distributions in up- and down-regulated, and transcription unchanged transcripts in the Δ*0076* mutant. The box indicates the range from the lower to the upper quartile, and the line inside each box refers to the median length of 5′UTRs. Extreme outliers are depicted by dots.
(TIF)

S6 Fig The 5′UTR structures predicted by Mfold. Hairpin structures like transcription terminator in the 5′UTRs of some down-regulated transcripts in the Δ*0076* mutant (SI Fig. S4-5′UTR structures) predicted by Mfold software (unafold.rna.albany.edu/?q=mfold) were shown.
(PDF)

S7 Fig Cold shock effect on the expression of 5′UTR fused mCherry in *M*. *maripaludis* wild strain S0001 and TRAM0076 gene deletion mutant (Δ*0076*). The middle exponential 37°C cultures of the 5′UTR-mCherry reporter strains as used in Fig 5C were cold shocked at 4°C for the indicated times (x axis). mCherry expression was then measured as in Fig 5C. Averages of the mCherry fluorescence from triplicate cultures and the standard deviations are shown.
(TIF)

S8 Fig Agarose gel detection of the RNAs co-immunoprecipitated by Flag-tagged TRAM0076 (RIP1 and RIP2) and un-tagged TRAM0076 (mock) (A), and that from the Input samples (Input1 and Input2) (B).
(TIF)

S9 Fig Box-plot shows the FPKM distribution of Flag-tagged TRAM0076 co-immunoprecipitated RNAs. The box indicates the range from the lower to the upper quartile, and the line inside the box refers to the median value of **FPKM**. Extreme outliers are depicted by dots.
(TIF)

S1 Table Strains and plasmids used in this study. (DOCX)

S2 Table Primers used in this study. (DOCX)

S1 Dataset Comparison of gene expressions in *Methanococcus maripaludis* wild-type strain S0001 and the Δ*0076* mutant. (XLS)

S2 Dataset Transcript start sites (TSSs) identified in the 5′ end libraries of *Methanococcus maripladius* by differential RNA-seq. (XLSX)

S3 Dataset 3XFlag-tagged TRAM0076 co-immunoprecipitated RNAs (RIP) from the cells of *M*. *maripladius* S2. (XLSX)
