# Supplementary figures and images for: The archaeal RNA chaperone TRAM0076 shapes the transcriptome and optimizes the growth of Methanococcus maripaludis
Source: PLoS Genet. 2019 Aug 12;15(8):e1008328. doi: 10.1371/journal.pgen.1008328 (PMC6705878; doi:10.1371/journal.pgen.1008328)

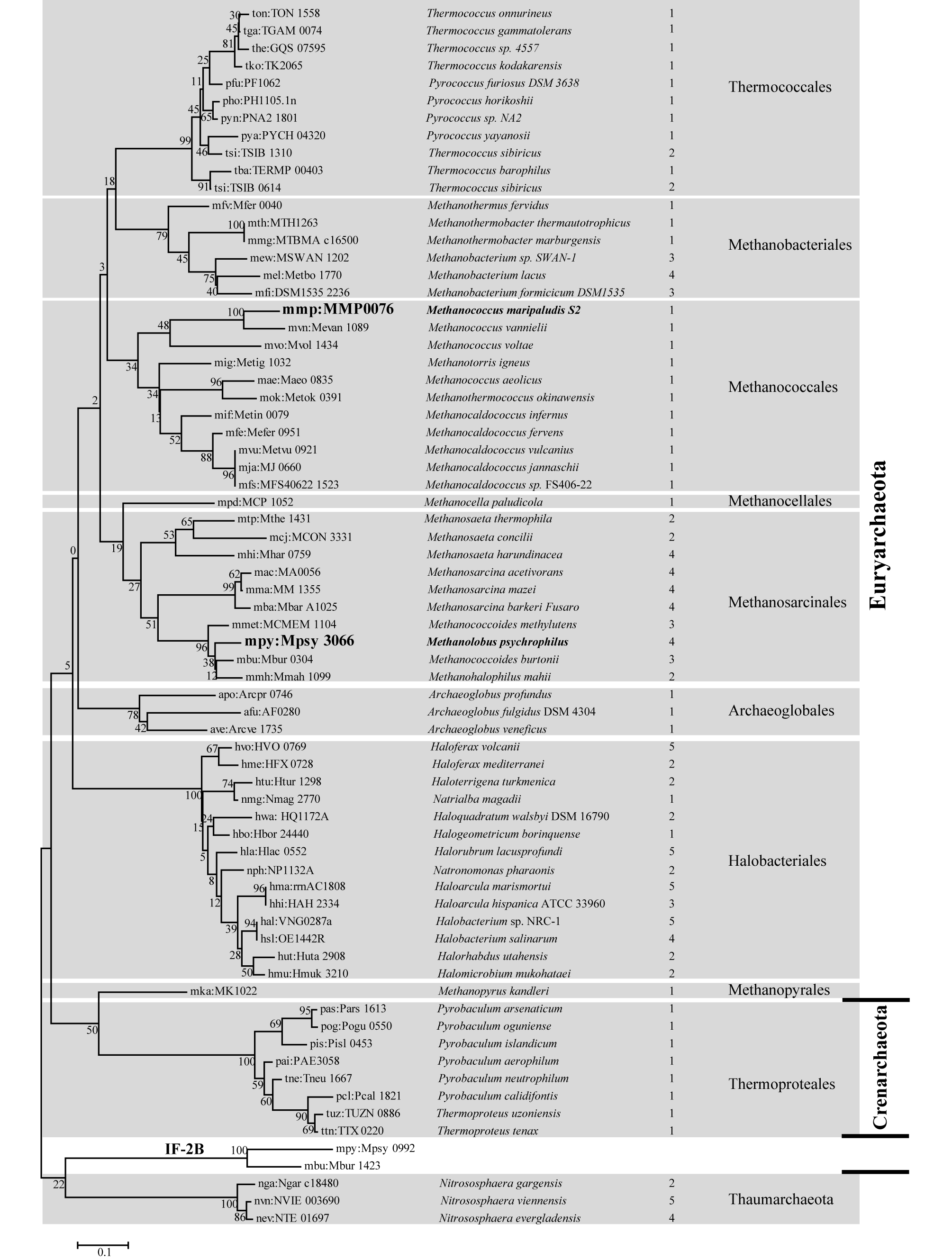

Supplement: S1 Fig — The tree was constructed using MEGA7.0, and the bar represents 10% sequence difference. The archaeal species and their encoded TRAM proteins are indicated, and numbers following each species are the TRAM genes copies. (TIF) [file pgen.1008328.s001.tif]

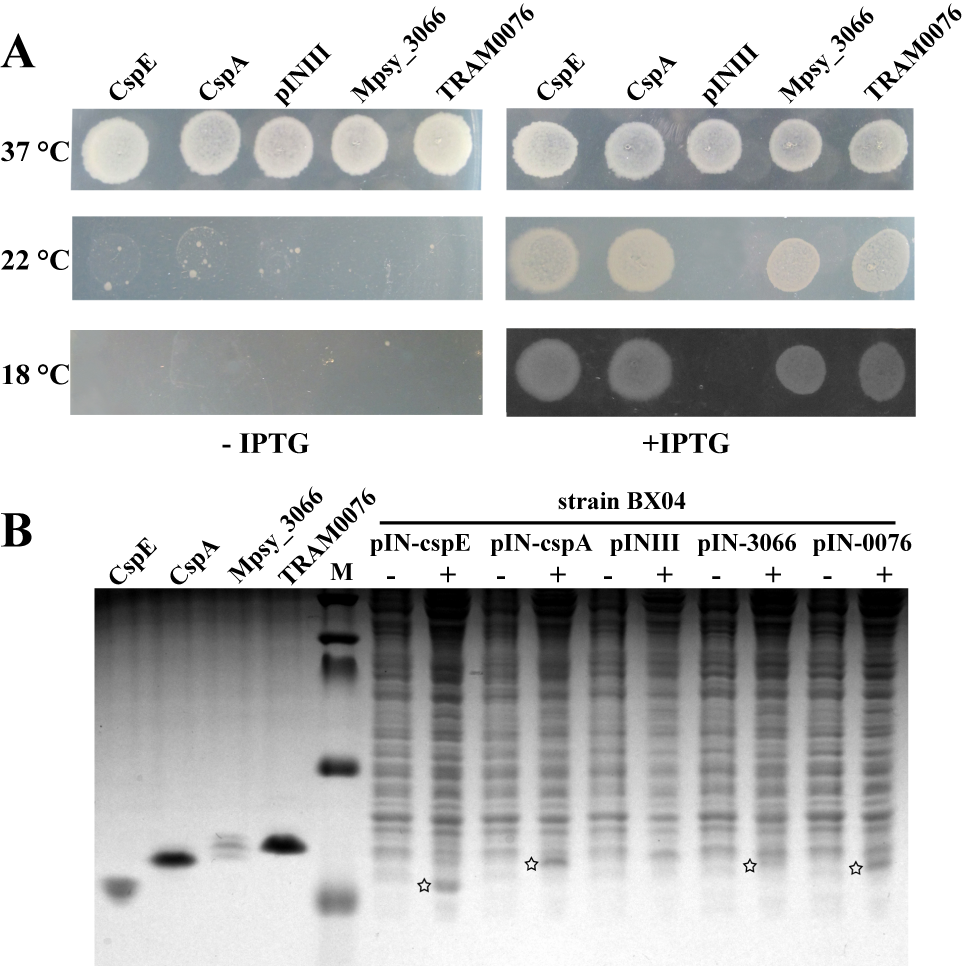

Supplement: S2 Fig — E. coli BX04 carries a deletion of the cspABGE operon, and its growth at low temperature is retarded. (A) Overnight liquid cultures of the E. coli strains that overexpress MMP0076, Mpsy_3066, cspA or cspE were diluted to OD600 of 0.9 with the fresh medium and then 10-fold serially diluted to 10−5 and spotted on LB plates supplemented with ampicillin. The plates were incubated at 18°C, 22°C and 37°C for 2-5 days with or without IPTG, as indicated. pINIII indicates the cultures carrying the empty plasmid vector. + and–, cultured in the presence or absence of IPTG, respectively. pINIII, strain carrying the empty plasmid pINIII. (B) SDS-PAGE confirmation of TRAM protein expression in strain BX04. Left: PAGE of 2.4 μg of purified CspA, CspE, TRAM3066 and TRAM0076. Right: Cell extracts from strain BX04 overexpressing cspA, cspE, Mpsy_3066 or MMP0076 were electrophoresed on 5% SDS-PAGE. + and–, cultured in the presence or absence of IPTG, respectively. pINIII, strain carrying the empty plasmid pINIII. Asterisks indicate the predicted migration of the overexpressed proteins. (TIF) [file pgen.1008328.s002.tif]

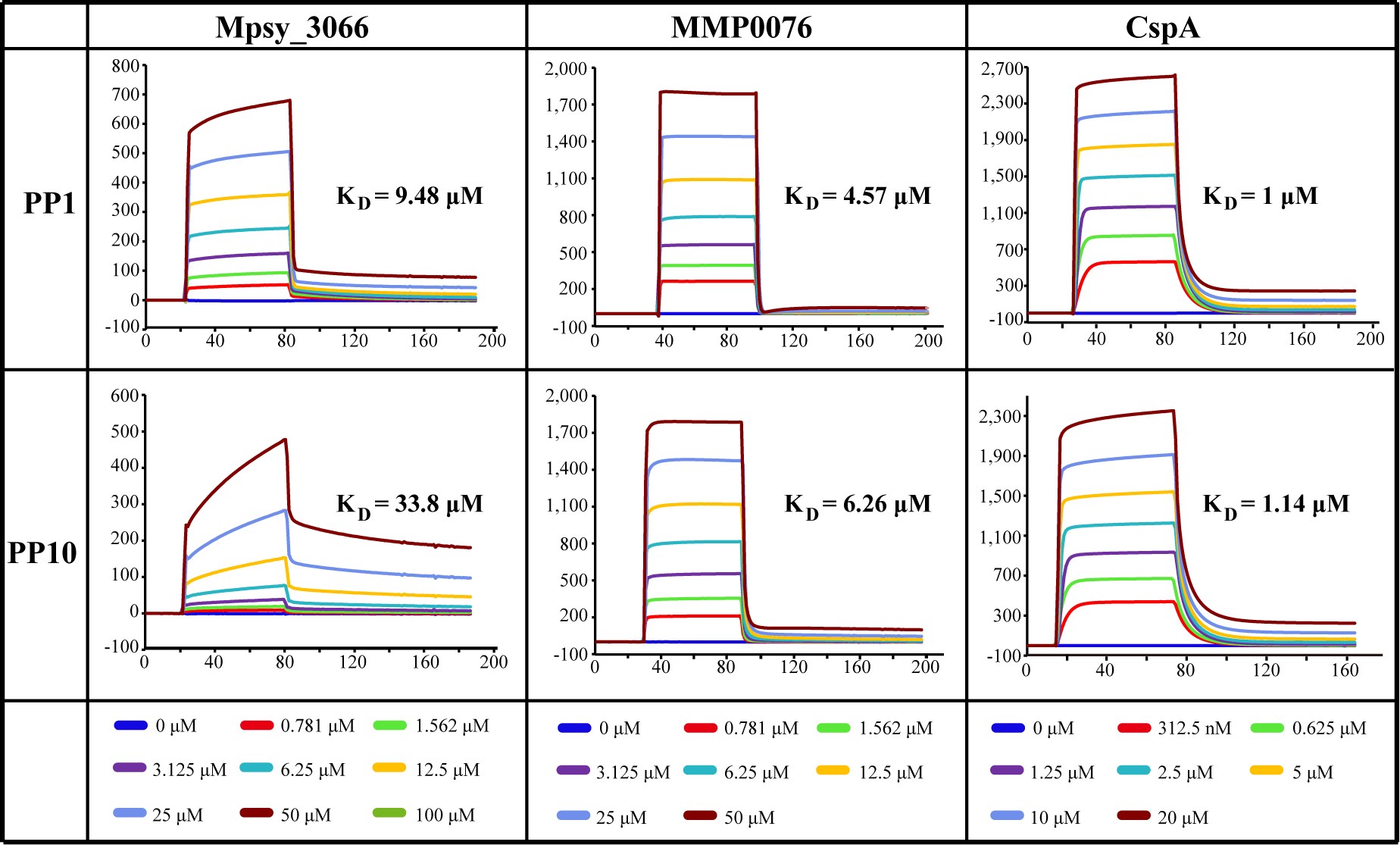

Supplement: S3 Fig — Two RNA Pentaprobes were used as substrates and immobilized to the SPR chip, and the RNA binding affinity was tested by addition of the indicated concentrations of TRAM0076. E. coli CspA and TRAM3066 are included as references. Calculated the equilibrium dissociation constant KD values are shown for each pair of protein-RNAs. The protein concentrations for each reaction are color coded and labeled in the bottom row. The x axis indicates the reaction time in seconds, and the y axis shows arbitrary resonance units (RU), where 1000 RU corresponds to a surface density of 1 ng/mm2. (TIF) [file pgen.1008328.s003.tif]

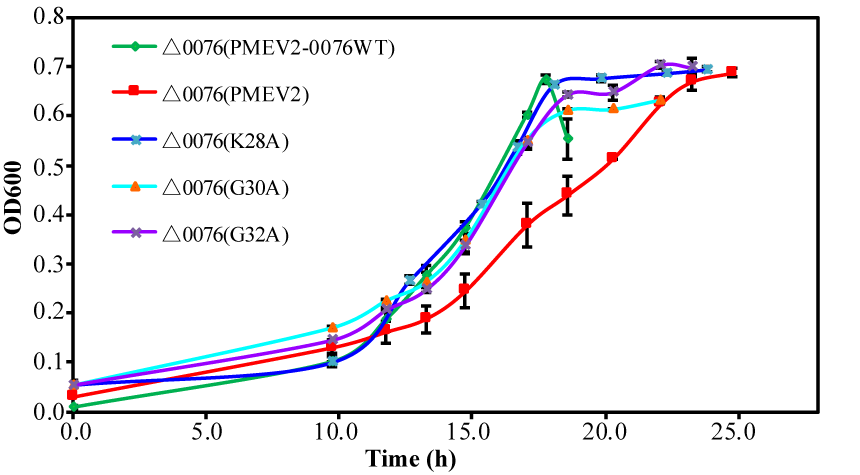

Supplement: S4 Fig — Complementation of the wild-type MMP0076 (PMEV2-0076WT) was included as the reference. Growth was measured at 37°C for triplicate cultures. Averages and standard deviations are shown. (TIF) [file pgen.1008328.s004.tif]

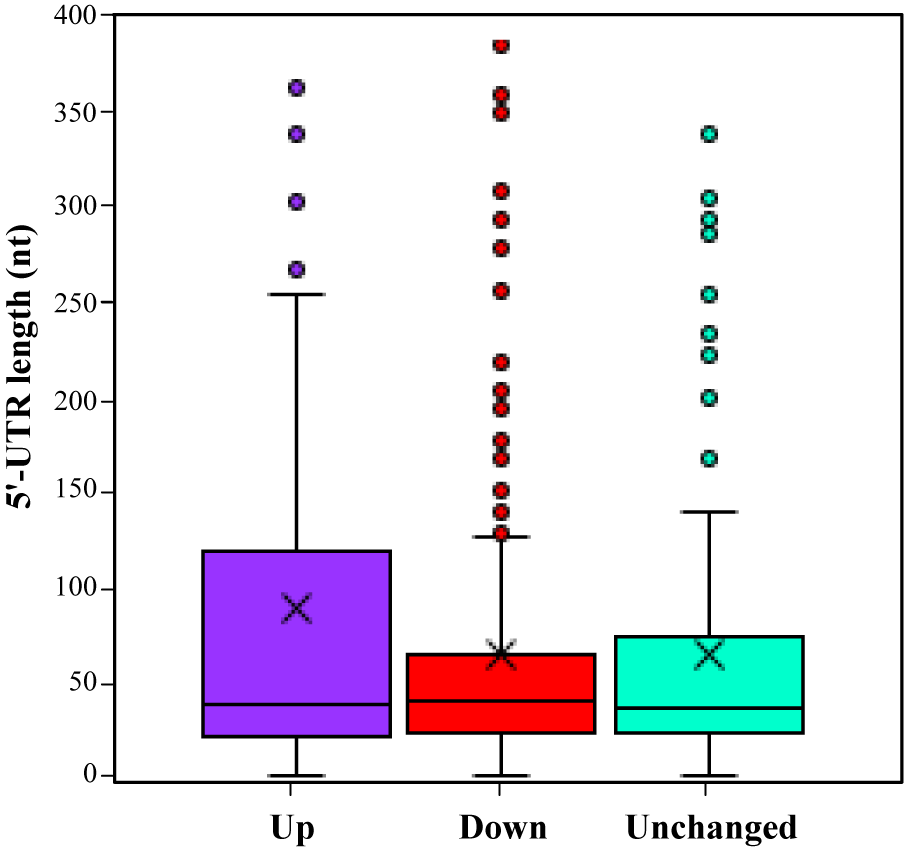

Supplement: S5 Fig — The box indicates the range from the lower to the upper quartile, and the line inside each box refers to the median length of 5′UTRs. Extreme outliers are depicted by dots. (TIF) [file pgen.1008328.s005.tif]

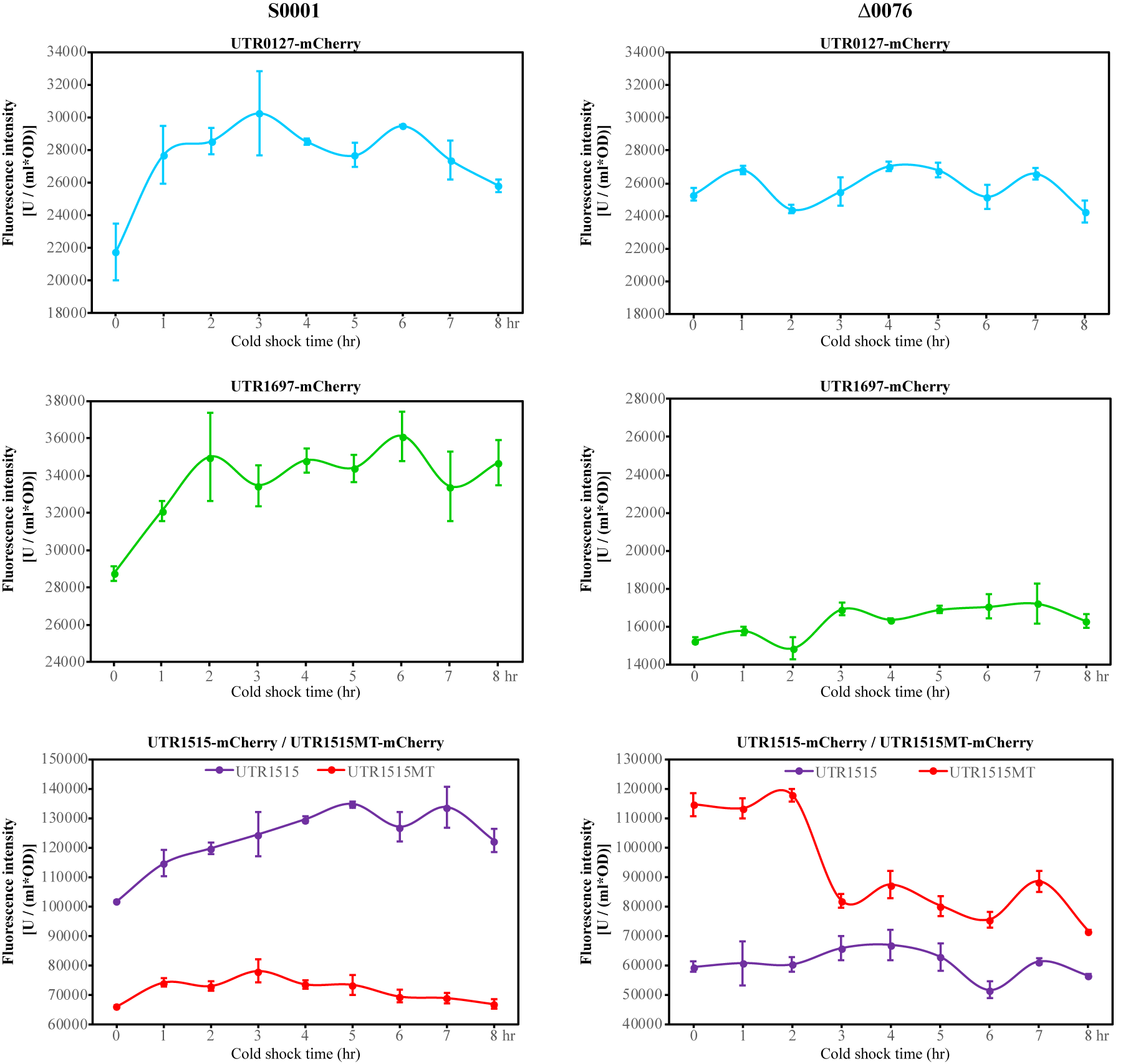

Supplement: S7 Fig — The middle exponential 37°C cultures of the 5′UTR-mCherry reporter strains as used in Fig 5C were cold shocked at 4°C for the indicated times (x axis). mCherry expression was then measured as in Fig 5C. Averages of the mCherry fluorescence from triplicate cultures and the standard deviations are shown. (TIF) [file pgen.1008328.s007.tif]

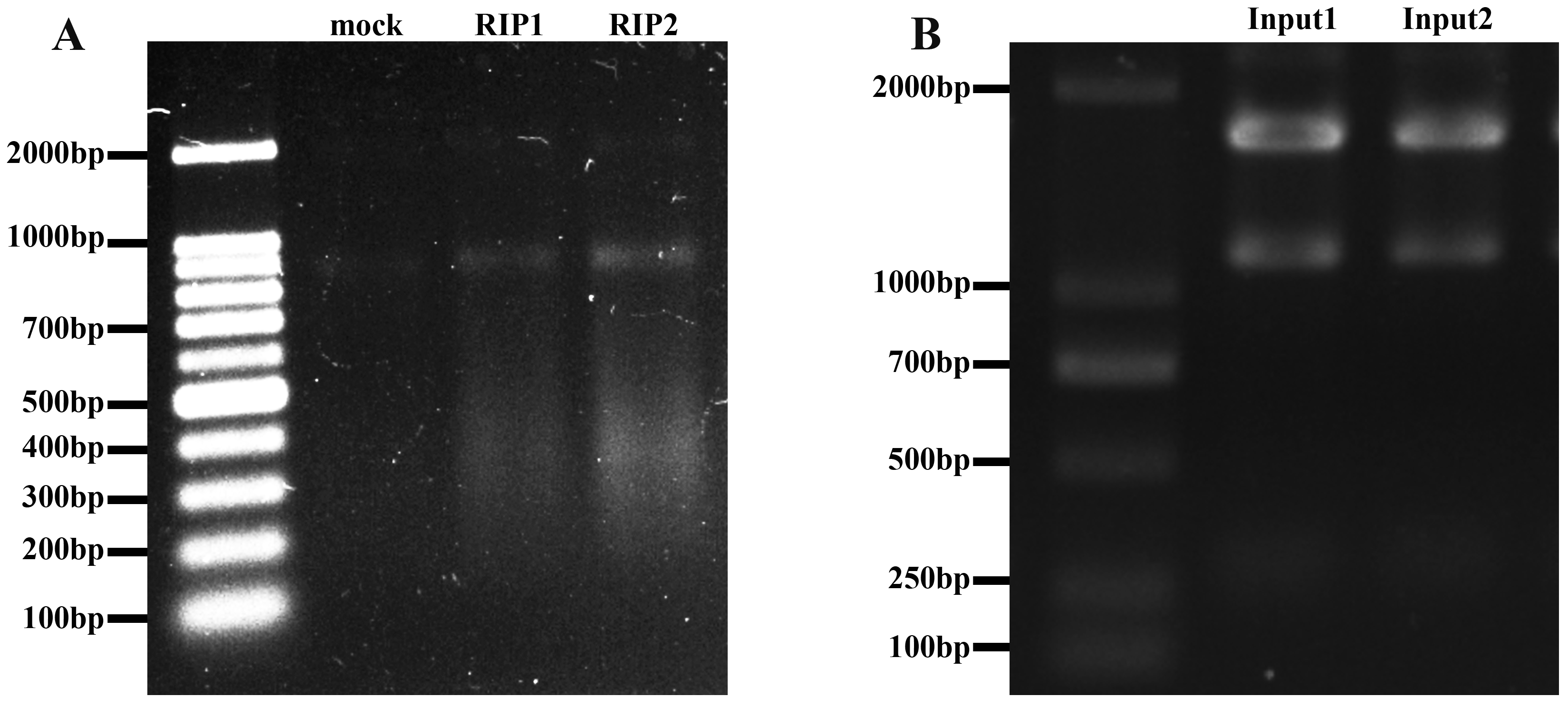

Supplement: S8 Fig — Agarose gel detection of the RNAs co-immunoprecipitated by Flag-tagged TRAM0076 (RIP1 and RIP2) and un-tagged TRAM0076 (mock) (A), and that from the Input samples (Input1 and Input2) (B). (TIF) [file pgen.1008328.s008.tif]

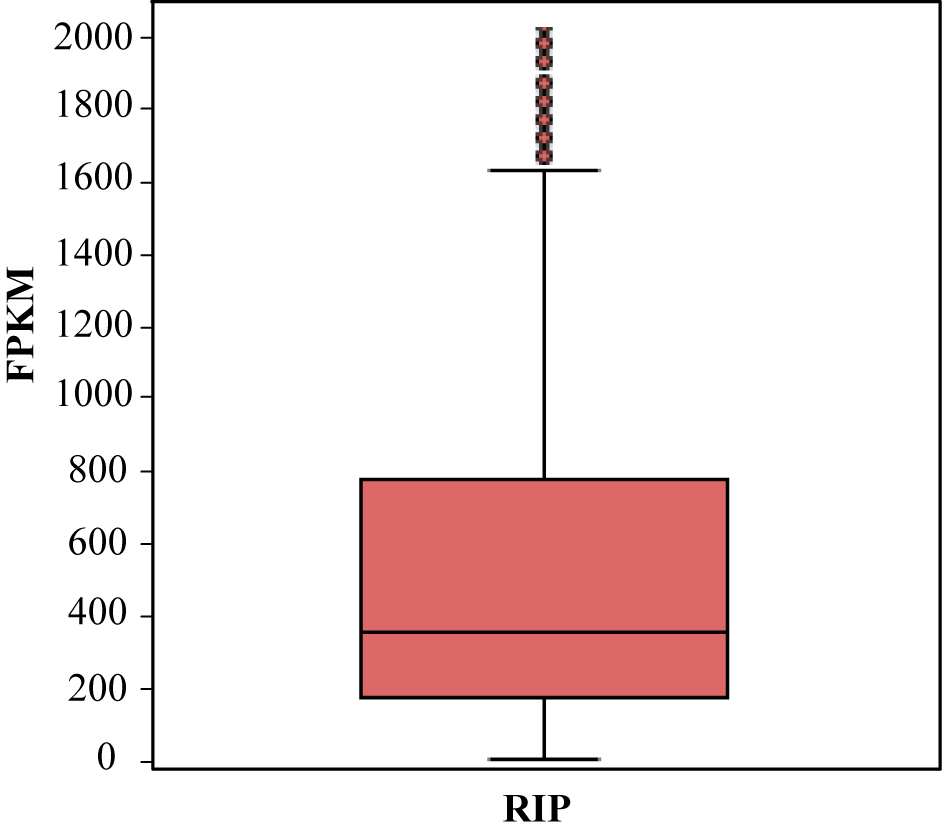

Supplement: S9 Fig — The box indicates the range from the lower to the upper quartile, and the line inside the box refers to the median value of FPKM. Extreme outliers are depicted by dots. (TIF) [file pgen.1008328.s009.tif]
