# Supplementary material for: The archaeal RNA chaperone TRAM0076 shapes the transcriptome and optimizes the growth of Methanococcus maripaludis
Source: PLoS Genet. 2019 Aug 12;15(8):e1008328. doi: 10.1371/journal.pgen.1008328 (PMC6705878; doi:10.1371/journal.pgen.1008328)
Supplement: S6 Fig — Hairpin structures like transcription terminator in the 5′UTRs of some down-regulated transcripts in the Δ0076 mutant (SI Fig. S4-5′UTR structures) predicted by Mfold software (unafold.rna.albany.edu/?q=mfold) were shown. (PDF) [file pgen.1008328.s006.pdf]

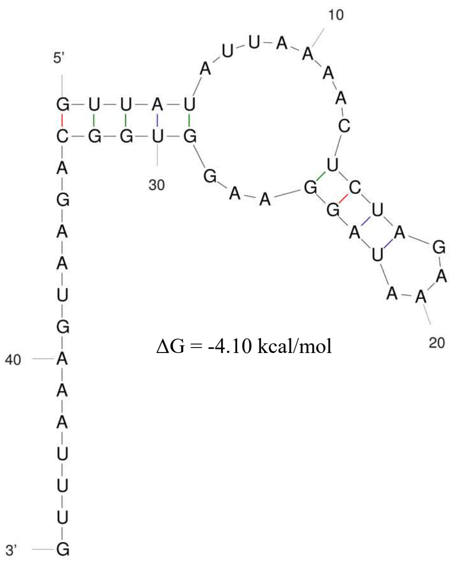

MMP0058

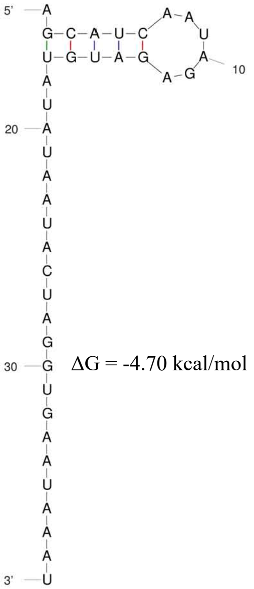

MMP0076

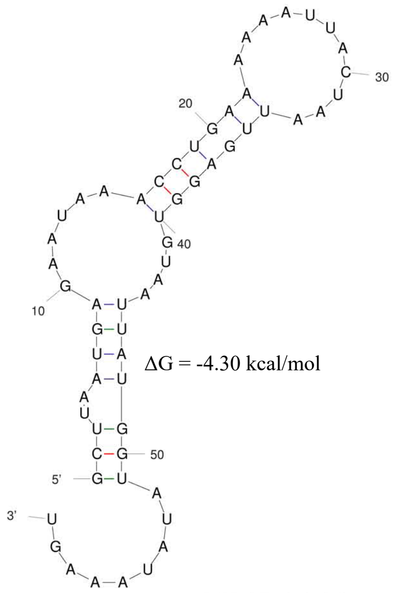

MMP0098

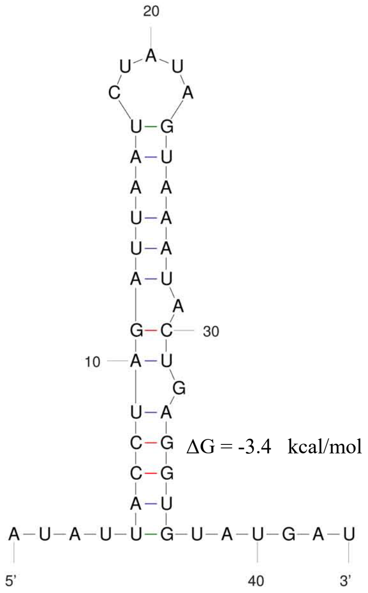

MMP0127

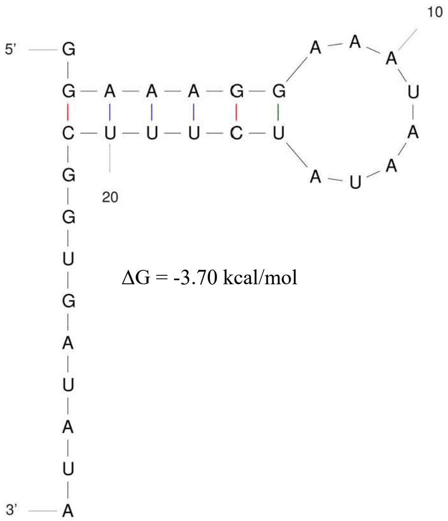

MMP0372

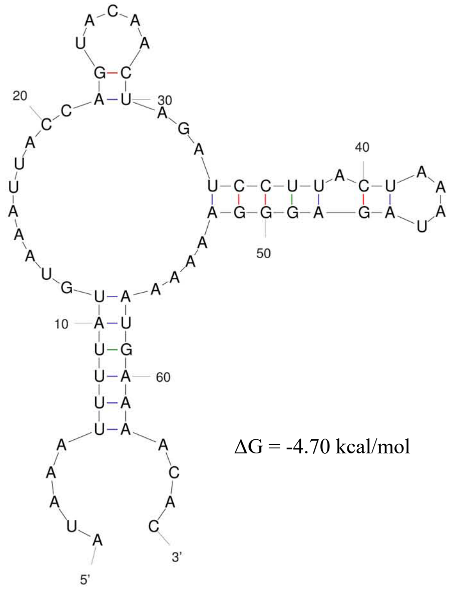

MMP0414

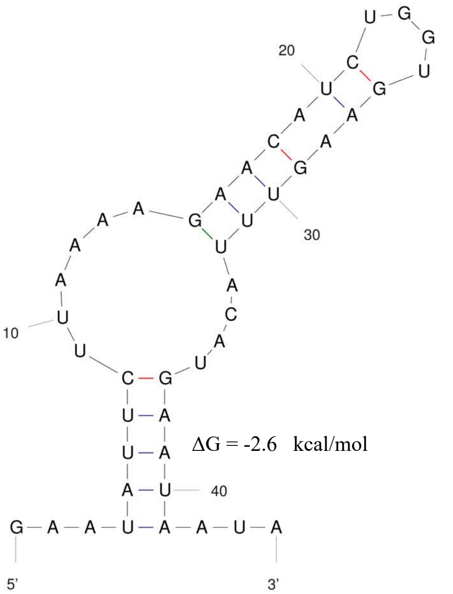

MMP0550

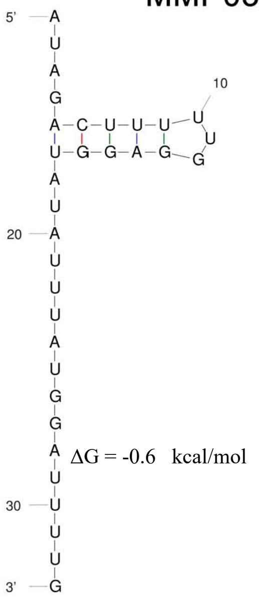

MMP0679

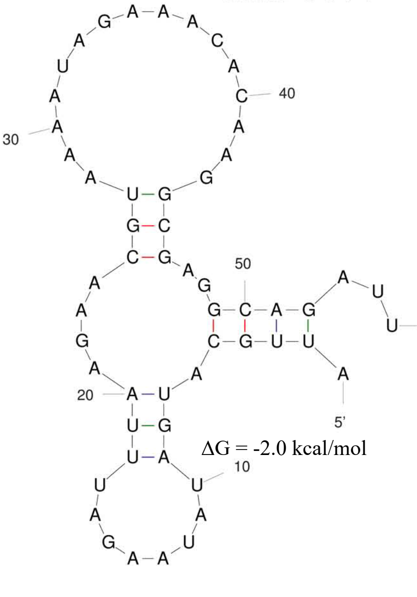

MMP1026

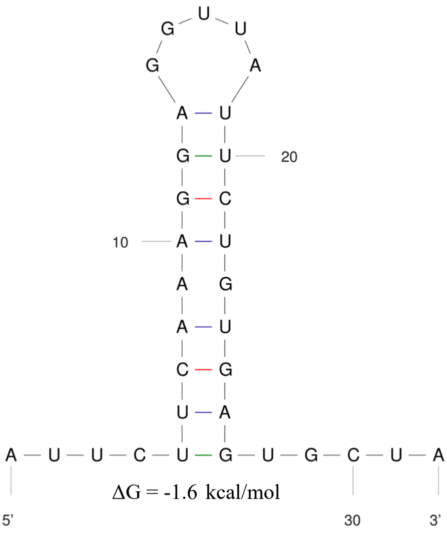

MMP1038

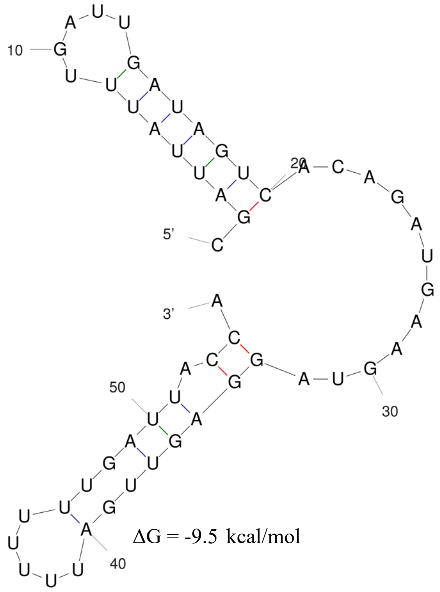

MMP1190

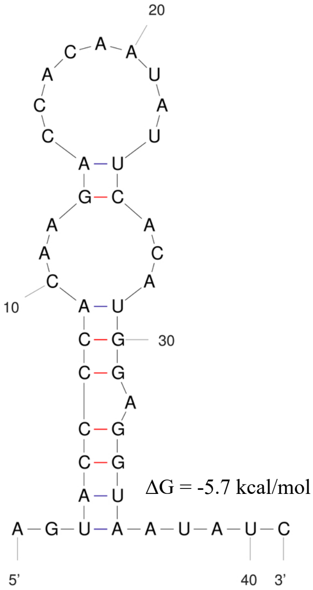

MMP1301

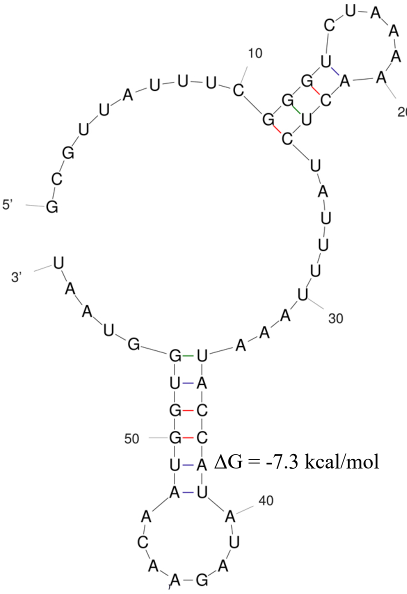

MMP1373

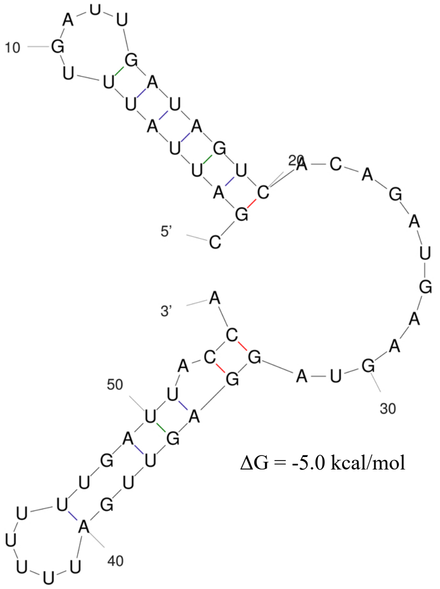

MMP1513

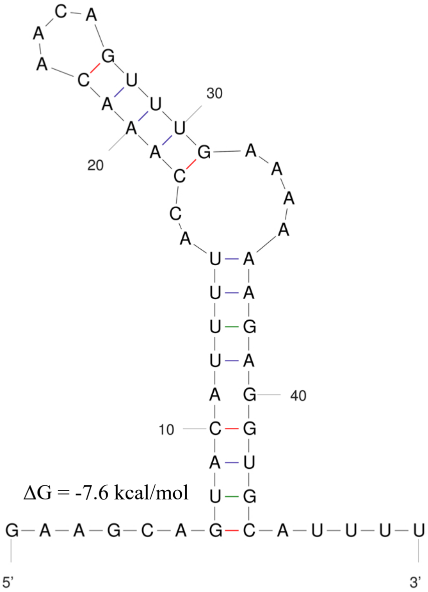

MMP1515

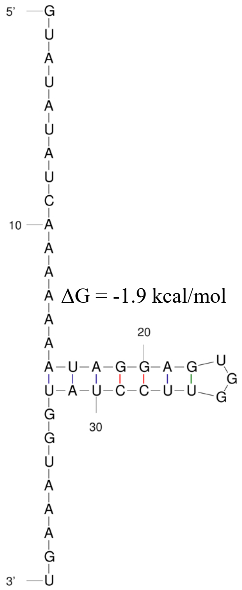

MMP1555

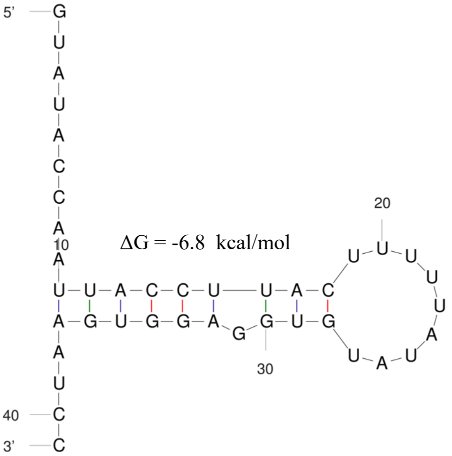

MMP1697

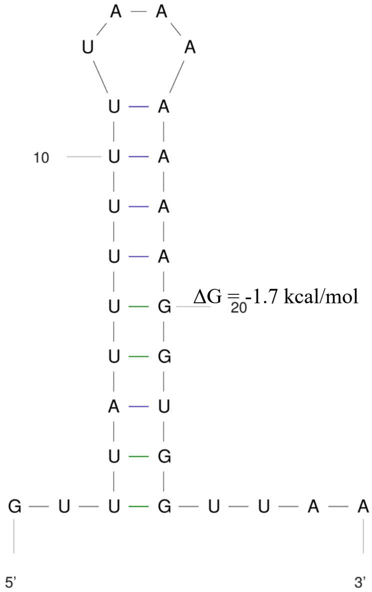

MMP1716

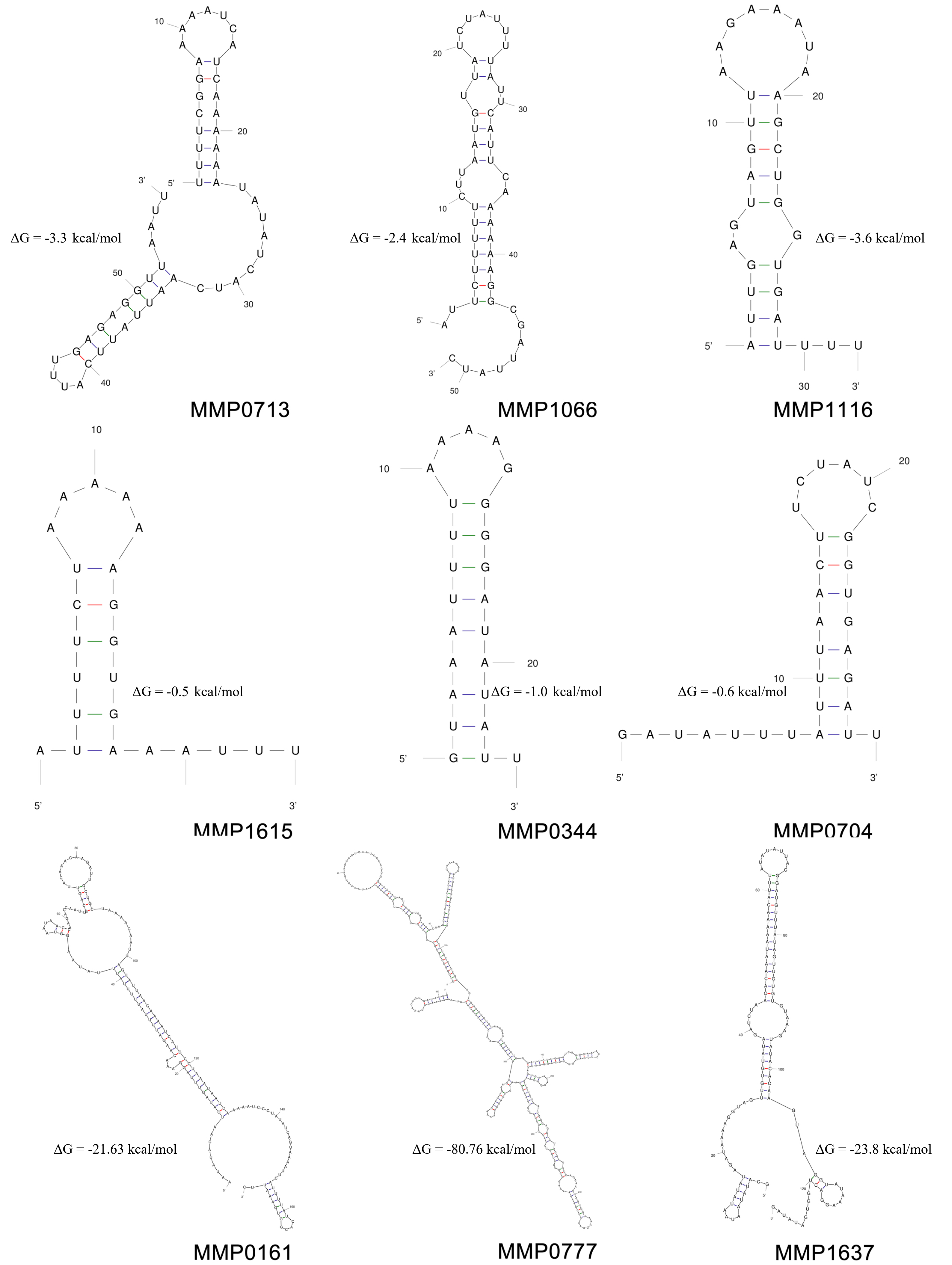
ΔG = -3.3 kcal/mol
MMP0713
ΔG = -2.4 kcal/mol
MMP1066
ΔG = -3.6 kcal/mol
MMP1116
ΔG = -0.5 kcal/mol
MMP1615
ΔG = -1.0 kcal/mol
MMP0344
ΔG = -0.6 kcal/mol
MMP0704
ΔG = -21.63 kcal/mol
MMP0161
ΔG = -80.76 kcal/mol
MMP0777
ΔG = -23.8 kcal/mol
MMP1637

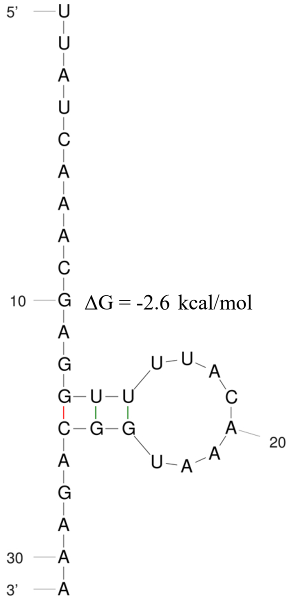

MMP0816

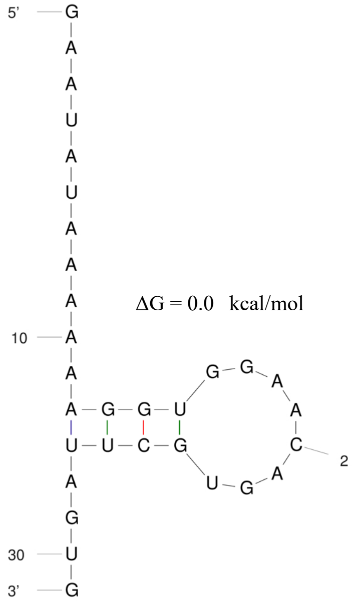

MMP1191

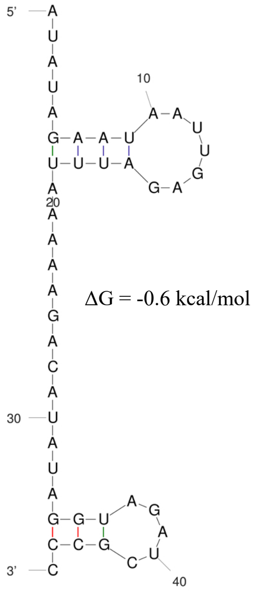

MMP0204

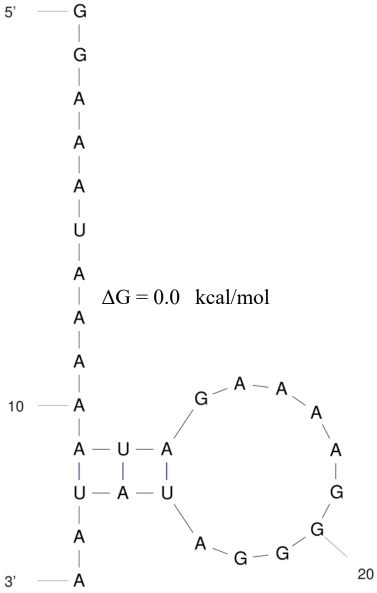

MMP0624

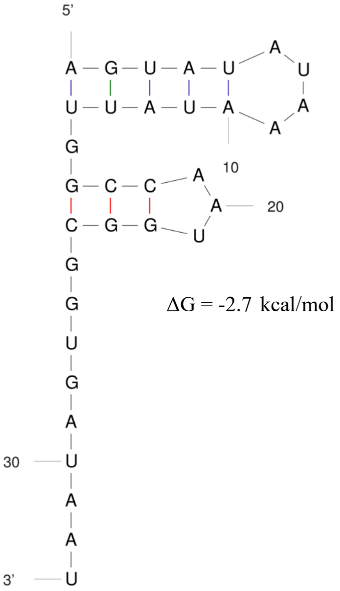

MMP0677

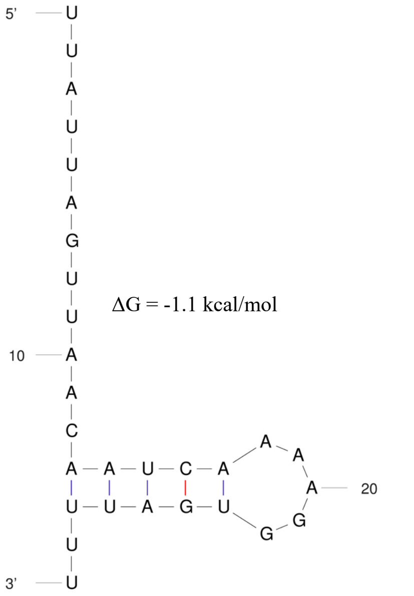

MMP0898

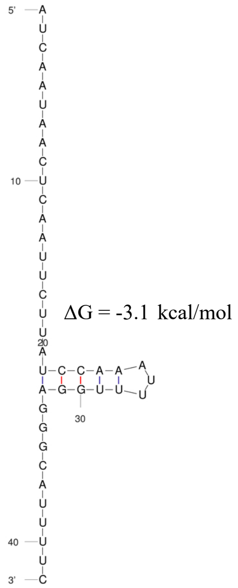

MMP1134

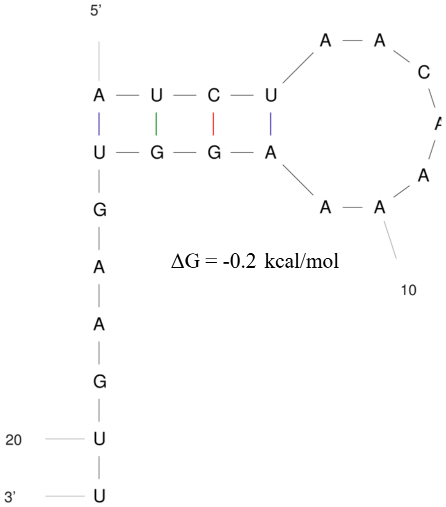

MMP1222

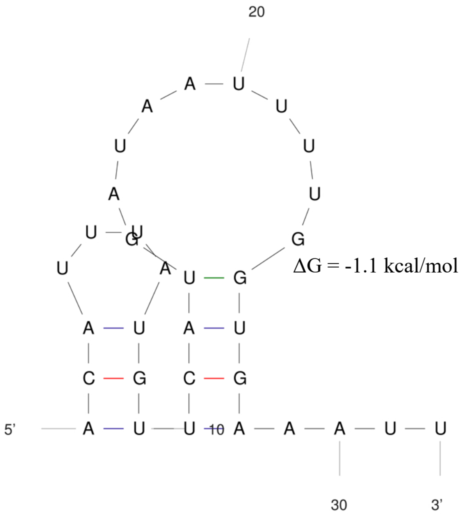

MMP1630
