# Supplementary material for: The archaeal RNA chaperone TRAM0076 shapes the transcriptome and optimizes the growth of Methanococcus maripaludis
Source: PLoS Genet. 2019 Aug 12;15(8):e1008328. doi: 10.1371/journal.pgen.1008328 (PMC6705878; doi:10.1371/journal.pgen.1008328)
Supplement: S1 Table — (DOCX) [file pgen.1008328.s010.docx]

**Table S1. Strains and plasmids used in this study**

| **Strains and plasmids** | **Characteristics and descriptions** | | **Reference or sources** | | |
| --- | --- | --- | --- | --- | --- |
| **Strains** |  | |  | | |
| *E. coli* DH5α | F^-^φ80d *lac*ZΔM15 Δ(*lac*ZYA-*arg* F) U169 *end*A1 *rec*A1 *hsd*R17(r_k_^-^,m_k_^+^) *sup*E44λ- *thi* -1 *gyr*A96 *rel*A1 *pho*A | Transgene, Beijing | |  |  |
| *E. coli* BL21 (DE3)pLysS | F^-^ ompT hsdS_B_(r_B_ - m_B_ -) gal dcm(DE3)pLysS Cam^r^ | | Novagen, Madison | |  |
| *E. coli* BX04 | Four csp genes (*cspA*,*cspB*,*cspE*, *cspG*) deleted | | Xia et al., 2001 [[1](#_ENREF_1)] | |  |
| *E. coli* RL211 | ρ-independent *trp*L terminator followed by *cat* gene | | Landick et al., 1990 [[2](#_ENREF_2)] | |  |
| *M. maripaludis* S0001 | grows optimally at 37 °C, Pur^S^, Neo^S^ | | Walters et al., 2011 [[3](#_ENREF_3)] | |  |
| *M. maripaludis* Δ0076 | 0076::Pac, Pur^R^, S0001 with MMP0076 deletion | | This study | |  |
| *M. maripaludis* MMP0076 com | 0076::pac, pMEV2-0076, Neo^R^, Δ0076 with MMP0076 complement | | This study | |  |
| *M. maripaludis* S0001 MMP0076D25A com | 0076::pac, pMEV2-0076D25A, Neo^R^, Δ0076 with MMP0076D25A complement | | This study | |  |
| *M. maripaludis* S0001 MMP0076K28A com | 0076::pac, pMEV2-0076K28A, Neo^R^, Δ0076 with MMP0076K28A complement | | This study | |  |
| *M. maripaludis* S0001 MMP0076G30A com | 0076::pac, pMEV2-0076G30A, Neo^R^, Δ0076 with MMP0076G30A complement | | This study | |  |
| *M. maripaludis* S0001 MMP0076G32A com | 0076::pac, pMEV2-0076G32A, Neo^R^, Δ0076 with MMP0076G32A complement | | This study | |  |
| *M. maripaludis* S0001 MMP0076I33A com | 0076::pac, pMEV2-0076I33A, Neo^R^, Δ0076 with MMP0076I33A complement | | This study | |  |
| *M. maripaludis* S0001 MMP0076R35A com | 0076::pac, pMEV2-0076R35A, Neo^R^, Δ0076 with MMP0076R35A complement | | This study | |  |
| *M. maripaludis* S0001 MMP0076F39A com | 0076::pac, pMEV2-0076F39A, Neo^R^, Δ0076 with MMP0076F39A complement | | This study | |  |
| *M. maripaludis* S0001 MMP0076F42A com | 0076::pac, pMEV2-0076F42A, Neo^R^, Δ0076 with MMP0076F42A complement | | This study | |  |
| *M. maripaludis* S0001 MMP0076K62A com | 0076::pac, pMEV2-0076K62A, Neo^R^, Δ0076 with MMP0076K62A complement | | This study | |  |
| *M. maripaludis* S0001 MMP0076F63A com | 0076::pac, pMEV2-0076F63A, Neo^R^, Δ0076 with MMP0076F63A complement | | This study | |  |
| *M. maripaludis* S0001 MMP0076F65A com | 0076::pac, pMEV2-0076F65A, Neo^R^, Δ0076 with MMP0076F65A complement | | This study | |  |
| *M. maripaludis* S0001 (pMEV4-0127PUO) | Pur^S^, pMEV4-0127PUO, Neo^R^ | | This study | |  |
| *M. maripaludis* S0001 (pMEV4-1515PUO) | Pur^S^, pMEV4-1515PUO, Neo^R^ | | This study | |  |
| *M. maripaludis* S0001 (pMEV4-1697PUO) | Pur^S^, pMEV4-1697PUO, Neo^R^ | | This study | |  |
| *M. maripaludis* S0001Δ0076 (pMEV4-0127PUO) | 0076::Pac, Pur^R^, S0001 with MMP0076 deletion, pMEV4-0127PUO, Neo^R^ | | This study | |  |
| *M. maripaludis* S0001Δ0076 (pMEV4-1515PUO) | 0076::Pac, Pur^R^, S0001 with MMP0076 deletion, pMEV4-1515PUO, Neo^R^ | | This study | |  |
| *M. maripaludis* S0001Δ0076 (pMEV4-1697PUO) | 0076::Pac, Pur^R^, S0001 with MMP0076 deletion, pMEV4-1697PUO, Neo^R^ | | This study | |  |
|  |  | |  | | |
| **Plasmids** |  | |  | | |
| pINIII | Amp^R^ | | Xia et al., 2001 [[1](#_ENREF_1)] | | |
|  |  | |  | | |
| pIN-cspE | pINIII with *E. coli cspE* inserted between NdeI and BamHI, Amp^R^ | | This study | | |
| pIN-cspA | pINIII with *E. coli cspA* inserted between NdeI and BamHI, Amp^R^ | | This study | | |
| pIN-3066 | pINIII with *Mpsy_3066* inserted between NdeI and BamHI, Amp^R^ | | This study | | |
| pIN-0076 | pINIII with *MMP0076* inserted between NdeI and BamHI, Amp^R^ | | This study | | |
| pIN-0076D25A | pINIII with *MMP0076D25A* inserted between NdeI and BamHI, Amp^R^ | | This study | | |
| pIN-0076K28A | pINIII with *MMP0076K28A* inserted between NdeI and BamHI, Amp^R^ | | This study | | |
| pIN-0076G30A | pINIII with *MMP0076G30A* inserted between NdeI and BamHI, Amp^R^ | | This study | | |
| pIN-0076G32A | pINIII with *MMP0076G32A* inserted between NdeI and BamHI, Amp^R^ | | This study | | |
| pIN-0076I33A | pINIII with *MMP0076I33A* inserted between NdeI and BamHI, Amp^R^ | | This study | | |
| pIN-0076R35A | pINIII with *MMP0076R35A* inserted between NdeI and BamHI, Amp^R^ | | This study | | |
| pIN-0076F39A | pINIII with *MMP0076F39A* inserted between NdeI and BamHI, Amp^R^ | | This study | | |
| pIN-0076F42A | pINIII with *MMP0076F42A* inserted between NdeI and BamHI, Amp^R^ | | This study | | |
| pIN-0076K62A | pINIII with *MMP0076K62A* inserted between NdeI and BamHI, Amp^R^ | | This study | | |
| pIN-0076F63A | pINIII with *MMP0076F63A* inserted between NdeI and BamHI, Amp^R^ | | This study | | |
| pIN-0076F65A | pINIII with *MMP0076F65A* inserted between NdeI and BamHI, Amp^R^ | | This study | | |
| pET28a | Kan^R^ | | Novagen, Madison | | |
| p28a-cspA | pET28a with *E. coli cspA* inserted between NcoI and XhoI, Kan^R^ | | This study | | |
| p28a-cspE | pET28a with *E. coli cspE* inserted between NcoI and XhoI, Kan^R^ | | This study | | |
| p28a-3066 | pET28a with *Mpsy_3066* inserted between NcoI and XhoI, Kan^R^ | | This study | | |
| p28a-0076 | pET28a with *MMP0076* inserted between NcoI and XhoI, Kan^R^ | | This study | | |
| pIJA03 | Pur^R^ | | Sarmiento et al. 2011[[4](#_ENREF_4)] | | |
| pIJA03-ΔMMP0076 | pIJA03 with *MMP0076* upstream fragment inserted between XhoI and BglII, and *MMP0076* downstream fragment inserted between KpnI and ClaI, Pur^R^ | | This study | | |
| pMEV2 | Neo^R^ | | Sarmiento et al. 2011[[4](#_ENREF_4)] | | |
| pMEV2-0076 | pMEV2 with MMP0076 inserted between NsiI/XbaI, Neo^R^ | | This study | | |
| pMEV2-0076D25A | pMEV2 with MMP0076D25A inserted between NsiI/XbaI, Neo^R^ | | This study | | |
| pMEV2-0076K28A | pMEV2 with MMP0076K28A inserted between NsiI/XbaI, Neo^R^ | | This study | | |
| pMEV2-0076G30A | pMEV2 with MMP0076G30A inserted between NsiI/XbaI, Neo^R^ | | This study | | |
| pMEV2-0076G32A | pMEV2 with MMP0076G32A inserted between NsiI/XbaI, Neo^R^ | | This study | | |
| pMEV2-0076I33A | pMEV2 with MMP0076I33A inserted between NsiI/XbaI, Neo^R^ | | This study | | |
| pMEV2-0076R35A | pMEV2 with MMP0076R35A inserted between NsiI/XbaI, Neo^R^ | | This study | | |
| pMEV2-0076F39A | pMEV2 with MMP0076F39A inserted between NsiI/XbaI, Neo^R^ | | This study | | |
| pMEV2-0076F42A | pMEV2 with MMP0076F42A inserted between NsiI/XbaI, Neo^R^ | | This study | | |
| pMEV2-0076K62A | pMEV2 with MMP0076K62A inserted between NsiI/XbaI, Neo^R^ | | This study | | |
| pMEV2-0076F63A | pMEV2 with MMP0076F63A inserted between NsiI/XbaI, Neo^R^ | | This study | | |
| pMEV2-0076F65A | pMEV2 with MMP0076F65A inserted between NsiI/XbaI, Neo^R^ | | This study | | |
| pMEV4 |  | | Lyu, et al., 2016 [[5](#_ENREF_5)] | | |
| pMEV4-mcherry-neo | pMEV4 with gene *pac* replaced with gene *neo* | | This study | | |
| pMEV4-0127PUO | pMEV4-mcherry-neo with upstream sequence of mcherry replaced by upstream sequence of *MMP0127* | | This study | | |
| pMEV4-1515PUO | pMEV4-mcherry-neo with upstream sequence of mcherry replaced by upstream sequence of *MMP1515* | | This study | | |
| pMEV4-1697PUO | pMEV4-mcherry-neo with upstream sequence of mcherry replaced by upstream sequence of *MMP1697* | | This study | | |
| pMEV4-1515PUOMT | pMEV4-mcherry-neo with upstream sequence of mcherry replaced by mutated upstream sequence of *MMP1515* | | This study | | |
|  |  | |  | | |

**References**:

1. Xia B, Ke H, Inouye M. (2001) Acquirement of cold sensitivity by quadruple deletion of the *cspA* family and its suppression by PNPase S1 domain in *Escherichia coli*. Mol Microbiol 40: 179-88.

2. Landick R, Stewart J, Lee DN. (1990) Amino-acid changes in conserved regions of the beta-subunit of *Escherichia coli* RNA polymerase alter transcription pausing and termination. Gene Dev 4: 1623-36. doi: Doi 10.1101/Gad.4.9.1623.

3. Walters AD, Smith SE, Chong JPJ. (2011) Shuttle vector system for *Methanococcus maripaludis* with improved transformation efficiency. Appl Environ Microbiol 77: 2549-51. doi: 10.1128/aem.02919-10.

4. Sarmiento BF, Leigh JA, Whitrnan WB. (2011) Genetic systems for hydrogenotrophic methanogens. Method Enzymol 494: 43-73. doi: 10.1016/B978-0-12-385112-3.00003-2.

5. Lyu Z, Jain R, Smith P, Fetchko T, Yan YJ, Whitman WB. (2016) Engineering the autotroph *Methanococcus maripaludis* for geraniol production. ACS Synth Biol 5: 577-81. doi: 10.1021/acssynbio.5b00267.
