# Supplementary material for: The archaeal RNA chaperone TRAM0076 shapes the transcriptome and optimizes the growth of Methanococcus maripaludis
Source: PLoS Genet. 2019 Aug 12;15(8):e1008328. doi: 10.1371/journal.pgen.1008328 (PMC6705878; doi:10.1371/journal.pgen.1008328)
Supplement: S2 Table — (DOCX) [file pgen.1008328.s011.docx]

**Table S2. Primers used in this study**

| **Primers** | **Sequences (5’-3’) ^a^** | **Purposes** |
| --- | --- | --- |
| P1 | GGAATTCCATATGCAGTCAAGTGCTCCAGTAG | P1/P2- pIN-3066 |
| P2 | CGGGATCCTTAAGCAACTTCGCCAAA |  |
| P3 | GGAATTCCATATGGCTTTCGGTAA | P3/P4- pIN-0076 |
| P4 | CGCGGATCCTTATACTCTTTCTGCGA |  |
| P5 | GGAATTCCATATGTCCGGTAAAATGACT | P5/P6- pIN-cspA |
| P6 | CGCGGATCCTTACAGGCTGGTTACGTT |  |
| P7 | GGAATTCCATATGTCTAAGATTAAAG | P7/P8- pIN-cspE |
| P8 | CGCGGATCCTTACAGAGCGATTA |  |
| P9 | CATGCCATGGTGCAGTCAAGTGCTCCAG | P9/P10- p28a-3066 |
| P10 | CCGCTCGAGAGCAACTTCGCCAAAA |  |
| P11 | CATGCCATGGTGGCTTTCGGTA | P11/P12- p28a-0076 |
| P12 | CCGCTCGAGTACTCTTTCTGCGA |  |
| P13 | CATGCCATGGTGTCCGGTAAAATGACTG | P13/P14- p28a-cspA |
| P14 | CCGCTCGAGCAGGCTGGTTACGTTA |  |
| P15 | CATGCCATGGTGTCTAAGATTAAAG | P15/P16- p28a-cspE |
| P16 | CCGCTCGAGCAGAGCGATTAC |  |
| P17 | CCGCTCGAGACATTACATCCACATACT | P17/P18-*MMP0076* upstream fragment |
| P18 | GAAGATCTATTTATTCACCTAGTATT |  |
| P19 | CGGGGTACCTTTCACGTCTTCCCTTAA | P19/P20-*MMP0076* downstream fragment |
| P20 | CCCATCGATGCGTAGATGCTGAAGTTT |  |
| P21 | CCAATGCATGGCTTTCGGTAAAC | P21/P22-pMEV2-0076 |
| P22 | GCTCTAGATTATACTCTTTCTGCGA |  |
| P23 | GGCTTTACACTTTATGCTTCC | P23/P24-for sequencing the pINIII derived plasmids |
| P24 | CGAGCTGCGTCATCTTTA |  |
| P25 | TTCAGGGAGCTCGAGATAAGAATTACTAGT | P25/P26-For sequencing the pMEV2 derived plasmids |
| P26 | TCGGCAGGAGCAAGGTGAGA |  |
| P27 | AATATGAAGTAACAATTGAA**GCC**ATGGGTAAAGGTGGA | P27/P28- pIN-0076D25A, pMEV2-0076D25A |
| P28 | TCCACCTTTACCCATGGCTTCAATTGTTACTTCATATT |  |
| P29 | CAATTGAAGACATGGGT**GCA**GGTGGAGACGGAATC | P29/P30- pIN-0076D25A, pMEV2-0076D25A |
| P30 | GATTCCGTCTCCACCTGCACCCATGTCTTCAATTG |  |
| P31 | GACATGGGTAAAGGT**GCA**GACGGAATCGCTAGA | P31/P32- pIN-0076D25A, pMEV2-0076D25A |
| P32 | TCTAGCGATTCCGTCTGCACCTTTACCCATGTC |  |
| P33 | GGTAAAGGTGGAGAC**GCA**ATCGCTAGAATCGATG | P33/P34- pIN-0076D25A, pMEV2-0076D25A |
| P34 | CATCGATTCTAGCGATTGCGTCTCCACCTTTACC |  |
| P35 | TAAAGGTGGAGACGGA**GCC**GCTAGAATCGATGG | P35/P36- pIN-0076D25A, pMEV2-0076D25A |
| P36 | CCATCGATTCTAGCGGCTCCGTCTCCACCTTTA |  |
| P37 | GGAGACGGAATCGCT**GCA**ATCGATGGATTCGTTG | P37/P38- pIN-0076D25A, pMEV2-0076D25A |
| P38 | CAACGAATCCATCGATTGCAGCGATTCCGTCTCC |  |
| P39 | CGCTAGAATCGATGGA**GCC**GTTGTTTTCGTACCTA | P39/P40- pIN-0076D25A, pMEV2-0076D25A |
| P40 | TAGGTACGAAAACAACGGCTCCATCGATTCTAGCG |  |
| P41 | TCGATGGATTCGTTGTT**GCC**GTACCTAACGCAGAA | P41/P42- pIN-0076D25A, pMEV2-0076D25A |
| P42 | TTCTGCGTTAGGTACGGCAACAACGAATCCATCGA |  |
| P43 | TTACCGCTGTAAAAGAA**GCA**TTCGCTTTCGCAGAAAG | P43/P44- pIN-0076D25A, pMEV2-0076D25A |
| P44 | CTTTCTGCGAAAGCGAATGCTTCTTTTACAGCGGTAA |  |
| P45 | ACCGCTGTAAAAGAAAAA**GCC**GCTTTCGCAGAAAG | P45/P46- pIN-0076D25A, pMEV2-0076D25A |
| P46 | CTTTCTGCGAAAGCGGCTTTTTCTTTTACAGCGGT |  |
| P47 | GTAAAAGAAAAATTCGCT**GCC**GCAGAAAGAGTATAA*G* | P47/P48- pIN-0076D25A, pMEV2-0076D25A |
| P48 | CTTATACTCTTTCTGCGGCAGCGAATTTTTCTTTTAC |  |
| P49 | ATCAAAAAAAACTAGAGGAGACGACCTTCCATGATTGAACAAGATGGATTG | P49/P50- Neo fragment for pMEV4-neo |
| P50 | CGGGTGGCTTCGGTCGGAGCCATCAGAAGAACTCGTCAAGAAG |  |
| P51 | TGATGGCTCCGACCGAAGCCACCCG | P51/P52- skeleton fragment for pMEV4-neo |
| P52 | GGAAGGTCGTCTCCTCTAGTTTTTTTTGATATATACATCATAAC |  |
| P53 | TACTAGATGAATTCCTCGAGAGCATCAGTTATTTCGCATAG | P53/P54- 5′ fragment for pMEV4-0127PUO |
| P54 | TTATCTTCTTCTCCTTTTGAAACCATTGAAAAGTTTGTGATTCCTG |  |
| P55 | TACTAGATGAATTCCTCGAGCTGGTTTAATGCCCCATG | P55/P56- 5′ fragment for pMEV4-1515PUO |
| P56 | TTATCTTCTTCTCCTTTTGAAACCATGTTTTCAGGTAAAACTCCTG |  |
| P57 | TACTAGATGAATTCCTCGAGTTACTCTACATCTCAAATAACTATTTTTAAAAG | P57/P58- 5′ fragment for pMEV4-1697PUO |
| P58 | TTATCTTCTTCTCCTTTTGAAACCATGACGAATACACCCACTTTG |  |
| P59 | ATGTTAGTTTAATATGTATCTTGATATGCTCTTC | P59/P60-For sequencing the pMEV4 derived plasmids |
| P60 | TGGTGGTCGAATGGGCAGGT |  |
| P61 | CCTCTTCGCTATTAC | general forward primer |
| P62 | TAAAGTAAGATAAGGCAAGACAAG | P61/P62 for PP1 template |
| P63 | TAGAATAGAGCGCAACTGAAGTCA | P61/P63 for PP2 template |
| P64 | ATGGGTAGGATAGGCGAGGC | P61/P64 for PP3 template |
| P65 | GGTGGGATTAGGGCAGCTCGAC | P61/P65 for PP4 template |
| P66 | CTCCAACCTCACACCAC | P61/P66 for PP5 template |
| P67 | CGGGATCCCGTACCATACTATAC | P61/P67 for PP6 template |
| P68 | CGGAATTCTACGA | P61/P68 for PP7 template |
| P69 | TATCTTACTTTAGTTTCATT | P61/P69 for PP8 template |
| P70 | CGCTCTATTCTACTGT | P61/P70 for PP9 template |
| P71 | TATCCTACCCATTG | P61/P71 for PP10 template |
| P72 | CCTAATCCCACCTA | P61/P72 for PP11 template |
| P73 | GTGAGGTTGGAGTCC | P61/P73 for PP12 template |
| P74 | 5’Bio-/rU/GGGTTTTTTTTTTTTTTTTTT | P74 used as the SPR linker |
| P75 | AAAAAAAAAAAAAAAAAACCC | P75 used as the SPR probe |
| P76 | TGTAAAACGACGGCCAGTGA | P76/P77 for PP1 and PP10 DNA templates containing A_18_C_3_ sequence |
| P77 | GCTATGACCATGATTACGCCAAG |  |
| P78 | CATACCGAACGTCATGAAGAAACCAAACAACAGTTTGAA | P78/P79-pMEV4-1515PUOMT |
| P79 | TTCAAACTGTTGTTTGGTTTCTTCATGACGTTCGGTATG |  |
| P80 | GACGGATCTCCAATTAATGCGGTT | P80/P81 qPCR stand-curve template of MMP0127 |
| P81 | AACCCTCTGAACTTGTGCCTGCTG |  |
| P82 | GCAAAAGAAGCAGGAATCCCAGAAAT | P82/P83 RIP-qPCR of MMP0127 |
| P83 | TCAGGCATTACTTTTTCAGCATCTCC |  |
| P84 | ACCTTGCAAACGATGGAAAAATT | P84/P85 qPCR stand-curve template of MMP1515 |
| P85 | TTGCCCGAAAAACCGAATACTT |  |
| P86 | GCAGGAAGAATTATCGCAGAAACAG | P86/P87 RIP-qPCR of MMP1515 |
| P87 | TACCGTCGCCAACTTCTTTTTCTT |  |
| P88 | ACTTTTTCTAATATTAATGGGCGCTA | P88/P89 qPCR stand-curve template of MMP1697 |
| P89 | AGCCACCACATACTTCCTCGTTAAC |  |
| P90 | TGCAGTGGATTGTGAAGCCGTAAAG | P90/P91 RIP-qPCR of MMP1697 |
| P91 | CGTGGATCTTAGGGGTGCATGCT |  |
| P92 | CCCTTGGAGACAGTGGCTTGATAA | P92/P93 qPCR stand-curve template of MMP1289 |
| P93 | GCTGCTGGTTAAATGCCGTTGT |  |
| P94 | TGGCACTAAGACCAGCAAGATGTTAC | P94/P95 RIP-qPCR of MMP1289 |
| P95 | GGATTCCAAAGCGTTGTGTCTTATTA |  |
| P96 | CCCCGCCATTTACTACCATTTACAC | P96/P97 qPCR stand-curve template of MMP1579 |
| P97 | TGGATCACGCGGCATACTTTG |  |
| P98 | TGGCAAGATTACACTCAGGAAAAAG | P98/P99 RIP-qPCR of MMP1579 |
| P99 | CGCTTTTGCCGGTGATTAATTTTAC |  |
| P100 | CCGCTTGAACACATTGCTCCA | P100/P101 qPCR stand-curve template of MMP1360 |
| P101 | TTCAAGGTAAAGCGGTGCAGAGTAT |  |
| P102 | TGGTTCCGACACATGAGATTATTCC | P102/P103 RIP-qPCR of MMP1360 |
| P103 | CTGCGGTAGGGCTCATTCTGGTT |  |
| P104 | TGCCAGGTAGAGTCTTTCAATCGTTT | P104P105 qPCR stand-curve template of MMP0076 |
| P105 | TGTTCTGAAATTGCCGGATCCAT |  |
| P106 | GGCTTTCGGTAAACCCGCAATGA | P106/P107 RIP-qPCR of MMP0076 |
| P107 | CGATTCTAGCGATTCCGTCTCCAC |  |
| P108 | GGCGGTACCCTCCTAAAAATGCAT | P108/P109 qPCR stand-curve template of RNase P RNA |
| P109 | CCCGGTGTCCACTAACTTCAACGT |  |
| P110 | GAAGCTCTGCCCACCCAATTGT | P110/P111 RIP-qPCR of RNase P RNA |
| P111 | GCCTGCCTTCTGTAATTTCGGTGA |  |
| P112 | AGCCCTCCTGCGATTCATTAAGTACT | P112/P113 qPCR stand-curve template of 16s rRNA |
| P113 | CCCCGCGTTCCCAATCCTTA |  |
| P114 | TGGGGGATAACCTTGGGAAACTGAG | P114/P115 RIP-qPCR of 16s rRNA |
| P115 | GGCCGTGTCTCAGTCCCCATCTC |  |

a. Bold letters represent the mutation sites for the key residue mutagenesis of TRAM0076.
